# Unlocking the puzzle: non-defining mutations in SARS-CoV-2 proteome may affect vaccine effectiveness

**DOI:** 10.3389/fpubh.2024.1386596

**Published:** 2024-08-15

**Authors:** Eugenia Ulzurrun, Ana Grande-Pérez, Daniel del Hoyo, Cesar Guevara, Carmen Gil, Carlos Oscar Sorzano, Nuria E. Campillo

**Affiliations:** ^1^Center for Biological Research Margarita Salas, Spanish National Research Council (CSIC), Madrid, Spain; ^2^National Center for Biotechnology, Spanish National Research Council (CSIC), Madrid, Spain; ^3^Institute of Mathematical Sciences, Spanish National Research Council (CSIC), Madrid, Spain; ^4^Department of Cellular Biology, Genetics, and Physiology, University of Malaga, Málaga, Spain; ^5^Mechatronics and Interactive Systems - MIST Research Center, Universidad Tecnológica Indoamérica, Quito, Ecuador

**Keywords:** SARS-CoV-2, mutations, proteome, vaccine, conservation

## Abstract

**Introduction:**

SARS-CoV-2 variants are defined by specific genome-wide mutations compared to the Wuhan genome. However, non-clade-defining mutations may also impact protein structure and function, potentially leading to reduced vaccine effectiveness. Our objective is to identify mutations across the entire viral genome rather than focus on individual mutations that may be associated with vaccine failure and to examine the physicochemical properties of the resulting amino acid changes.

**Materials and methods:**

Whole-genome consensus sequences of SARS-CoV-2 from COVID-19 patients were retrieved from the GISAID database. Analysis focused on Dataset_1 (7,154 genomes from Italy) and Dataset_2 (8,819 sequences from Spain). Bioinformatic tools identified amino acid changes resulting from codon mutations with frequencies of 10% or higher, and sequences were organized into sets based on identical amino acid combinations.

**Results:**

Non-defining mutations in SARS-CoV-2 genomes belonging to clades 21 L (Omicron), 22B/22E (Omicron), 22F/23A (Omicron) and 21J (Delta) were associated with vaccine failure. Four sets of sequences from Dataset_1 were significantly linked to low vaccine coverage: one from clade 21L with mutations L3201F (ORF1a), A27- (S) and G30- (N); two sets shared by clades 22B and 22E with changes A27- (S), I68- (S), R346T (S) and G30- (N); and one set shared by clades 22F and 23A containing changes A27- (S), F486P (S) and G30- (N). Booster doses showed a slight improvement in protection against Omicron clades. Regarding 21J (Delta) two sets of sequences from Dataset_2 exhibited the combination of non-clade mutations P2046L (ORF1a), P2287S (ORF1a), L829I (ORF1b), T95I (S), Y145H (S), R158- (S) and Q9L (N), that was associated with vaccine failure.

**Discussion:**

Vaccine coverage associations appear to be influenced by the mutations harbored by marketed vaccines. An analysis of the physicochemical properties of amino acid revealed that primarily hydrophobic and polar amino acid substitutions occurred. Our results suggest that non-defining mutations across the proteome of SARS-CoV-2 variants could affect the extent of protection of the COVID-19 vaccine. In addition, alteration of the physicochemical characteristics of viral amino acids could potentially disrupt protein structure or function or both.

## 1 Introduction

Severe acute respiratory syndrome coronavirus 2 (SARS-CoV-2), first reported in Wuhan (China) in late 2019, soon spread around the globe. To date, more than 700 million people have been infected and 6.97 million have died worldwide. Despite the availability of vaccines, SARS-CoV-2 remains a cause for concern. Like other RNA viruses, SARS-CoV-2 is endowed with a high mutation rate and high viral load and accumulates mutations with each replication cycle. As a result, viral variants that differ in one or more nucleotides are continuously being generated in infected hosts (1–3). The nomenclature of SARS-CoV-2 variants plays a critical role in facilitating their clear identification, tracking, and global collaboration in understanding the evolutionary dynamics of the virus. For Nextstrain clade naming, a new major clade earns its designation once it attains a 20% frequency on a global scale, regardless of the time frame. When computing these frequencies, it is crucial to ensure a relatively uniform sampling of sequences across different times and geographical locations due to the considerable disparity in sequencing efforts among countries. Clade names are formulated using a standardized protocol, typically derived from the year of emergence and the subsequent available letter in the alphabet. Additionally, a newly identified clade must exhibit a minimum of two mutations differentiating it from its parent major clade. This systematic approach ensures consistency and accuracy in clade designation, facilitating the clear identification and tracking of viral lineages in genomic surveillance studies. SARS-CoV-2 has undergone several genetic mutations leading to the emergence of various clades since its first identification in 2019. A recent study using Belgian data revealed an enhanced immune escape capability exhibited by the Omicron variant compared to the Alpha and Delta variants, resulting in a substantial reduction in the protective efficacy conferred by both acquired immunity and vaccination. Furthermore, a decline in vaccine effectiveness over time was observed, underscoring the significance of booster doses to sustain long-term immunity (4).

In the Netherlands population, the effectiveness of primary and booster vaccination against SARS-CoV-2 infection was estimated overall and in four risk groups defined by age and medical conditions during the Delta and Omicron BA.1/BA.2 periods. The findings underscored the advantages of booster vaccinations in reducing infection rates, particularly within at-risk groups (5).

Investigations of the complete of SARS-CoV-2 proteome have often been limited to analyzing only a few sequences or individual sequences, rather than taking advantage of a large sequencing dataset. However, a direct RNA sequencing approach was employed to assess the SARS-CoV-2 transcriptome in Vero E6 cells, and mass spectrometry was used to explore the proteome and phosphoproteome of these virus-infected cells (6). Furthermore, a proteomic analysis of proteins extracted from nasopharyngeal swabs of 12 patients diagnosed with COVID-19 identified 13 different SARS-CoV-2 proteins. Additionally, host proteome analysis revealed that several key host proteins were uniquely expressed in patients with COVID-19 (7). In a separate study, distinct epitopes of seven different proteins were identified using the complete SARS-CoV-2 virus genome obtained from the NCBI database. However, the 12 protein sequences of the genome were formatted as FASTA files using RefSeq accessions (8). Furthermore, a proteome-wide study of SARS-CoV-2 assessed its potential to induce autoimmune diseases by segmenting the proteome into peptides and identifying shared peptides with experimentally confirmed human T-cell and B-cell epitopes (9).

The SARS-CoV-2 genome consists of a single-stranded, unsegmented, positive-polarity RNA molecule [(+) ssRNA] 29,903 nucleotides in length encoding 13 ORFs (10). Two-thirds of the viral genome corresponds to ORF1a and ORF1b, which express the two polyproteins pp1a and pp1ab, the latter though a − 1 ribosomal frameshift, and that are processed by two viral proteases into 16 non-structural proteins (nsp). ORF1a encodes nsp1 to nsp11 and ORF1b comprises nsp12 to nsp16. Non-structural proteins make up the replication and transcription machinery and are responsible for the maintenance of the viral genome (11). Some nsp proteins are targets for antiviral drugs such as nsp12, the RNA-dependent RNA polymerase (RdRp), and nsp5, the 3C-like protease (Mpro, 3CLpro) (12). In addition, nsp3, the papain-like protease (PLpro) is also a therapeutical target for antivirals (13).

The structural proteins are, namely, the surface glycoprotein or spike (S), the envelope protein (E), the membrane glycoprotein (M), and the nucleocapsid phosphoprotein (N). The S protein has been shown to play a major role in virus attachment and entry into cells, being a key antigen for development of vaccines and neutralizing antibodies, and as a pharmacological target (14–16). The E protein plays a key role in the pathogenesis of the virus affecting the binding of SARS-CoV-2 to the tight junction proteins (17). The M protein is responsible for maintaining the shape of the virion by spanning the membrane bilayer and facilitates budding of the viral particles from the host cells (18). Interestingly, the M protein was found to elicit IgM response during the acute phase of SARS-CoV-2 infection (19). The nucleocapsid function is to maintain the genome structure inside the envelope (20). The N protein has been identified as an important target for T-cell response, making it a suitable candidate for next-generation COVID-19 vaccines against emerging variants (21–23).

There are nine accessory proteins, ORF3a, 3d, 6, 7a, 7b, 8, 9b, 14, and 10, which are produced from the encoding accessory genes ORF3a, ORF3d, ORF6, ORF7a, ORF7b, ORF8, ORF9b, ORF14 and ORF10, respectively (24). Although the exact functions of SARS-CoV-2 accessory proteins remain to be determined, studies of other coronaviruses suggest that they are not essential for viral replication but can modulate replication and pathogenesis through interaction with host pathways including antiviral activation (25, 26) and viral translation (27).

The existing literature predominantly concentrates on isolated mutations within the S protein rather than examining mutations as a network involving multiple proteins. Individually, mutations in SARS-CoV-2 might not pose significant risks, however their collective effect in tandem with other mutations could amplify the virus’s transmissibility and virulence. Consequently, relying solely on information about distinct segments of the virus might provide an incomplete understanding. Therefore, a comprehensive study of mutations across the entire SARS-CoV-2 genome and proteome becomes essential. Such a holistic approach is critical in understanding the virus’s mechanisms for evading vaccines and is instrumental in the development of effective vaccines and therapies.

Mutations (including deletions) that alter protein sequence, may affect physicochemical properties and folding conformation of proteins resulting in changes in biological functions. Amino acid positions in proteins could be assigned a single conservation number based on the alignment of homologous proteins for quantification of amino acid substitutions (28, 29).

Given that structural variations due to mutations could affect vaccine effectiveness and drug function, and thus the severity of COVID-19, this work involved an extensive genome-wide mutation combination analysis of SARS-CoV-2 in vaccinated patients from Italian and Spanish populations as two independent datasets. Analyzing more than 7,500 proteomes from each population increases the statistical power and reliability of the results. Our objective was to identify mutations across the entire viral genome, rather than focusing on individual mutations, that may be associated with vaccine failure, in addition to analyze the physicochemical properties of the resulting amino acid changes. This study involved aligning SARS-CoV-2 genomic sequences from both vaccinated and unvaccinated COVID-19 patients using the Wuhan genome as reference. We examined the frequency of specific mutation sets and analyzed their physicochemical properties to understand how these mutations may affect the structure and function of viral proteins. This approach provides a comprehensive view of the genetic diversity of SARS-CoV-2 variants circulating in different geographic regions and contributes to a deeper understanding of the underlying mechanisms of the vaccine effectiveness, which is crucial for informing public health strategies and vaccine development efforts.

## 2 Materials and methods

### 2.1 SARS-CoV-2 genome sequences

Sequences of COVID-19 patients were retrieved from the Global Initiative on Sharing All Influenza Data (GISAID) (30): (a) Dataset_1 contained 7,154 aligned consensus sequences of SARS-CoV-2 genomes isolated from patients of Friuli-Venezia Giulia (Italy) from January 01, 2021 to June 24, 2023. Of these, 2,419 were fully vaccinated and 1,667 received a booster dose vaccination against the Omicron variant; (b) Dataset_2 contained 8,819 aligned genomes mainly from Catalonia (Spain) since January 01, 2021 to July 25, 2022. Of those, 2,969 were completely vaccinated and 699 received the third COVID-19 vaccine dose against Omicron. Table 1 shows a description of the datasets. Datasets are available in the Supplementary material.

**Table 1:** Brief description of Dataset_1 and Dataset_2.

	Dataset_1 (*n* = 7,154)	Dataset_2 (*n* = 8,819)
Female, *n* (%)	3,280 (45.8)	4,988 (56.6)
Male, *n* (%)	2,962 (41.4)	3,801 (43.1)
Unknown Gender, *n* (%)	912 (12.7)	30 (0.3)
< 65 years, *n* (%)	4,535 (63.4)	5,892 (66.8)
≥ 65 years, *n* (%)	1,684 (23.5)	2,927 (33.2)
Unknown Age, *n* (%)	935 (13.1)	0 (0.0)
Timeline	2021-01-01/2023-06-24	2021-01-01/2022-07-25
Fully Vaccinated, *n* (%)	2,419 (33.8)	2,969 (33.7)
Not Omicron/Omicron	322 (13.3)/2097 (86.7)	2,163 (72.9)/806 (27.2)
BioNTech-Pfizer	1,450 (59.9)	2,197 (74.0)
J&J-Janssen	60 (2.5)	94 (3.2)
Moderna-Lonza	827 (34.2)	154 (5.2)
Oxford-AstraZeneca	81 (3.3)	513 (17.2)
Unknown/Others	0 (0.0)/1 (0.1)	9 (0.3)/2 (0.1)
Booster, *n* (%)	1,667	699
Omicron	1,667	699
BioNTech-Pfizer	945 (56.7)	569 (81.4)
J&J-Janssen	12 (0.7)	3 (0.4)
Moderna-Lonza	710 (42.6)	40 (5.7)
Oxford-AstraZeneca	0 (0.0)	83 (11.9)
Unknown/Others	0 (0.0)/0 (0.0)	4 (0.6)/0 (0.0)
Doses Received, *n* (%)		
None	4,624 (64.6)	5,751 (65.2)
One dose	149 (2.1)	168 (1.9)
Two doses	694 (9.7)	2,160 (24.5)
Three doses	1,687 (23.6)	740 (8.4)
Main Variants, *n* (%)		
20I (Alpha)	656 (9.2)	261 (3.0)
20B	35 (0.5)	1 (0.0)
20E (EU1)	185 (2.6)	56 (0.6)
20A	81 (1.1)	11 (0.1)
21J (Delta)	2,166 (30.3)	4,527 (51.3)
21I (Delta)	97 (1.4)	117 (1.3)
21K (Omicron)	893 (12.5)	1770 (20.1)
21L (Omicron)	873 (12.2)	1,437 (16.3)
22B (Omicron)	999 (14.0)	538 (6.1)
22E (Omicron)	344 (4.8)	0 (0.0)
22F (Omicron)	176 (2.5)	0 (0.0)
23A (Omicron)	292 (4.1)	0 (0.0)

The NetAlign CLI software was used for sequence alignment (version 2.4.0) (31). Genome and coding sequences (CDS) of SARS-CoV-2 Wuhan-Hu-1 reference sequence NC_045512.2 were retrieved from GenBank. SARS-CoV-2 variants and mutations were sourced from the CoVariant website,^1^ which employs the Nextstrain nomenclature for identification of variants.

### 2.2 Mutations of interest

Changes in genome, including indeterminations during sequencing base calling, were referred to as non-synonymous or missense mutations when the codons contained mutations (substitutions or deletions) with a frequency of 10% or higher in the genomic sequence alignments. These were referred as mutation of interest (MOI). Sequences sharing the same combination of MOIs (haplotypes) were grouped together into set of sequences. Non-defining mutations were also referred to as additional MOIs.

### 2.3 Bioinformatic tools and statistical analysis

To identify amino acid changes within SARS-CoV-2 genomes, codon translation in coding sequences (CDS) was accomplished using scripts written in the R programming language (version 4.1.0) (32) and Python (version 3.8.11) (33). The Biopython library (version 1.76) (34) was employed for managing the amino acid alphabet. Qualitative variable analysis was performed using the Chi-square test from the scipy.stats Python module (scipy version 1.9.3) (35). We employed the trackViewer package (version 1.34.0) (36) for creating visual representations of mutations in SARS-CoV-2 proteins. Furthermore, EMBOSS Seqret (version 6.6.0.0) (37) was employed to record significant mutation combinations in mega non-interleaved output format. Data manipulation and analysis were carried out using libraries like Matplotlib (version 3.5.1) (38) and Pandas (version 1.2.3) (39). In-house scripts used in this study were developed by the authors. Scripts used in our analysis are openly available at: https://github.com/papersarscov2proteome/.

Undetermined amino acids and special characters from the Biopython dictionary (B, Z, J, U, and O) were represented as ‘X.’ Sets containing ‘X’ were excluded from further analysis, while those with a frequency of 1% or greater were retained for statistical examination. Statistical significance was determined with *p* values <0.05 (following False Discovery Rate (FDR) correction) (40). Vaccine coverage was identified when expected frequencies exceeded observed frequencies.

Genetic distances estimation within each sequence set was carried out using MEGA (41). The mean distance was calculated using the Bootstrap method for variance estimation, with 1,000 bootstrap replications, employing the p-distance model for amino acid substitution type. Ambiguous positions were eliminated for each sequence pair using the pairwise deletion option.

### 2.4 Patients

We categorized individuals as having complete active immunity or fully vaccinated (FV) as those who had received a minimum of 2 doses (or 1 dose in the case of the Janssen vaccine) at least 14 days prior to infection, regardless of the specific infectious variant. For FV patients who were infected by the Omicron variant, those who had received a third COVID-19 vaccine dose (or a second dose of Janssen vaccine) were designated as booster patients. Vaccines referred to as others include Sinovac and Sinopharm. Individuals who did not meet any of the above criteria were classified as not fully vaccinated.

### 2.5 Residue conservation

Conservation analysis stands as one of the most widely used methods for predicting functionally significant residues in protein sequences. Residue conservation, as defined by Livingstone et al. (29), employs two distinct methods to quantify a singular conservation score for each position. For both, the physicochemical properties assessed for the 20 amino acids take into account whether the molecules are hydrophobic, polar, small, proline, tiny, aliphatic, aromatic, positive, negative and charged. In this study, we used the method 1 which considers any property exhibiting positive or negative conservation. A deletion is considered to possess all of these properties for the conservation index calculation. In this work high conservation index refers around a range of 10–8, intermediate to 7 and 6, and values equal to or less than 5 with low conservation.

## 3 Results

### 3.1 Datasets description

**Figure 1:**
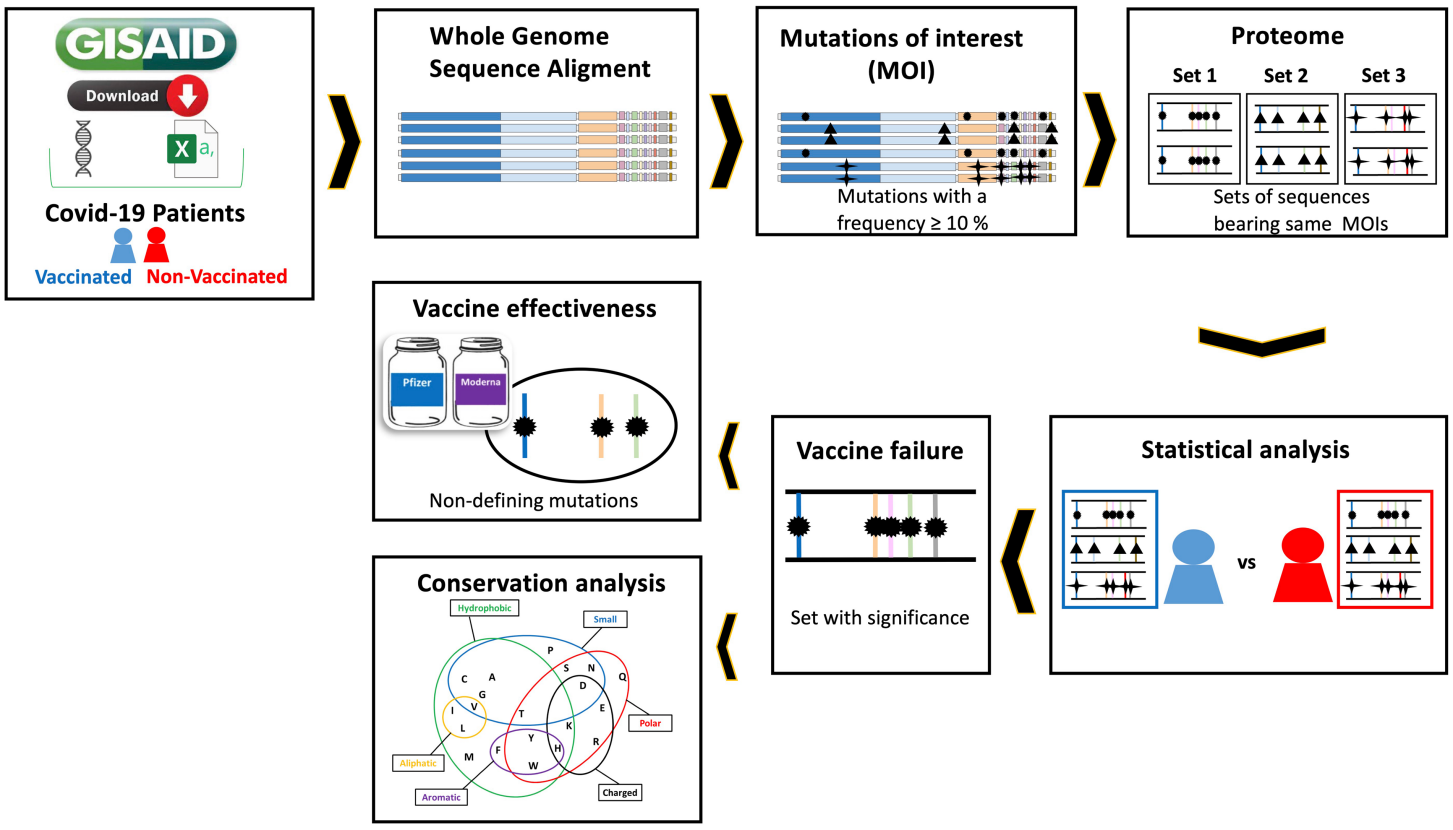
Flowchart of the methodology used in this study. 1. Sequences and metadata of vaccinated and unvaccinated COVID-19 patients from Italy and Spain were downloaded from GISAID. 2. The SARS-CoV-2 genome sequences of the COVID-19 patients were aligned to the Wuhan reference genome. 3. The mutations of interest (MOIs) are then defined. 4. Next, the proteomes are obtained and those sequences that have the same combination of MOIs are grouped together. 5 and 6. Statistical analysis is now performed using a Chi-square test to identify sets of sequences between fully vaccinated and unvaccinated patients that are associated with vaccine failure. 7. The role of non-clade-defining mutations in the risk of vaccine effectiveness is then investigated by comparing populations. 8. Finally, the physicochemical properties of the MOIs in the sequence sets associated with vaccine failure are analyzed.

For Dataset_1, we analyzed a total of 7,154 SARS-CoV-2 genome aligned consensus sequences. Out of these, 2,419 were from fully vaccinated (FV) COVID-19 patients, and 2097 had been infected with the Omicron variant (Table 1). In the FV group (comprising 2,419 out of 7,154 individuals), 59.9% had received the Pfizer vaccine, followed by 34.2% who had received the Moderna vaccine. A negligible proportion, less than 5%, had received the J&J-Janssen and Oxford-AstraZeneca vaccines. The predominant variant across several clades was Omicron, representing 50% of the total population (Table 1). Figure 1 illustrates the flowchart followed.

**Figure 2:**
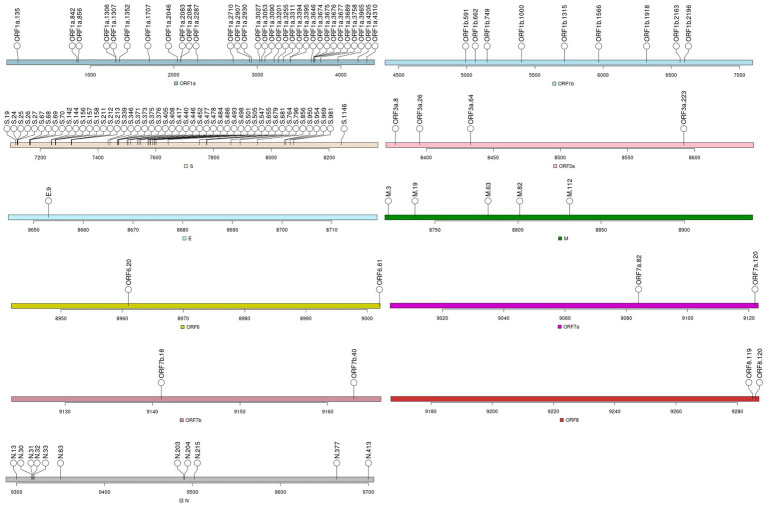
Mutations of interest (MOIs) identified for Dataset_1.

We identified 119 MOIs which combinations resulted in eight sets of sequences with a frequency greater than 1% ranging from Set1_ds1 to Set8_ds1 (Figure 2; Supplementary Figure S2A). For the sequence sets associated with vaccine failure, the timeline revealed a peak of cases belonging to clade 21L (Omicron) in May 2022, as shown by Set5_ds1. However, clades 22B and 22E of the Omicron variant exhibited two peaks, observed in Set6_ds1 and Set7_ds1 in September and December, respectively. By April 2023, the majority of infections were attributed to clades 22F and 23A of the Omicron variant. Detailed information on the clades associated with each set of sequences is provided in Tables 2, 3.

**Table 2:** Descriptive statistics of non-Omicron set of sequences within Dataset_1, comparing fully vaccinated and non-fully vaccinated COVID-19 patients.

	Set1_ds1 *n* = 817	Set2_ds1 *n* = 165	Set3_ds1 *n* = 327	Set4_ds1 *n* = 89
Full active immunity, *n*	182	1	3	3
FDR^a^ value	2.00E-13^b^	2.11E-19^b^	2.24E-37^b^	2.02E-10^b^
Clade	21J (Delta)	20A 20E (EU1)	20I (Alpha)	20I (Alpha) 20B
Peak^c^ (*n*)	December 2021 (231)	January 2021 (114)	March 2021 (120)	April 2021 (48)

^a^FDR, False discovery rate.^b^Expected frequency exceeded the observed frequency for fully vaccinated patients infected by SARS-CoV-2 sequences sharing the same combination of MOIs, in clinical terms this translates to vaccine coverage or efficacy.^c^Referred to the highest number of cases.

**Table 3:** Descriptive statistics of Omicron set of sequences of Dataset_1, comparing fully vaccinated and booster doses with non-fully vaccinated COVID-19 patients.

	Set5_ds1 *n* = 176	Set6_ds1 *n* = 453	Set7_ds1 *n* = 179	Set8_ds1 *n* = 294
Full active immunity, *n*	144	384	155	259
FDR^a^ value	1.58E-41	1.32E-122	1.12E-50	1.24E-88
Booster, *n*	118	341	139	228
FDR value	3.20E-31	1.18E-118	2.21E-50	4.88E-83
Clade	21L (Omicron)	22B (Omicron) 22E (Omicron)	22B (Omicron) 22E (Omicron)	22F (Omicron) 23A (Omicron)
Peak^b^ (*n*)	May 2022 (108)	September 2022 (109)	December 2022 (52)	April 2023 (94)

^a^FDR, False discovery rate.^b^Referred to the highest number of cases.

For Dataset_2, we analyzed aligned consensus sequences from 8,819 COVID-19 patients. Of these, 2,969 were from fully vaccinated patients with 2,163 of them infected with non-Omicron variants (Table 1). Pfizer was the most frequently administered vaccine (74.0%), followed by AstraZeneca (17.3%). Infection with the 21J (Delta) variant occurred in 51.3% of cases (Table 1).

**Figure 3:**
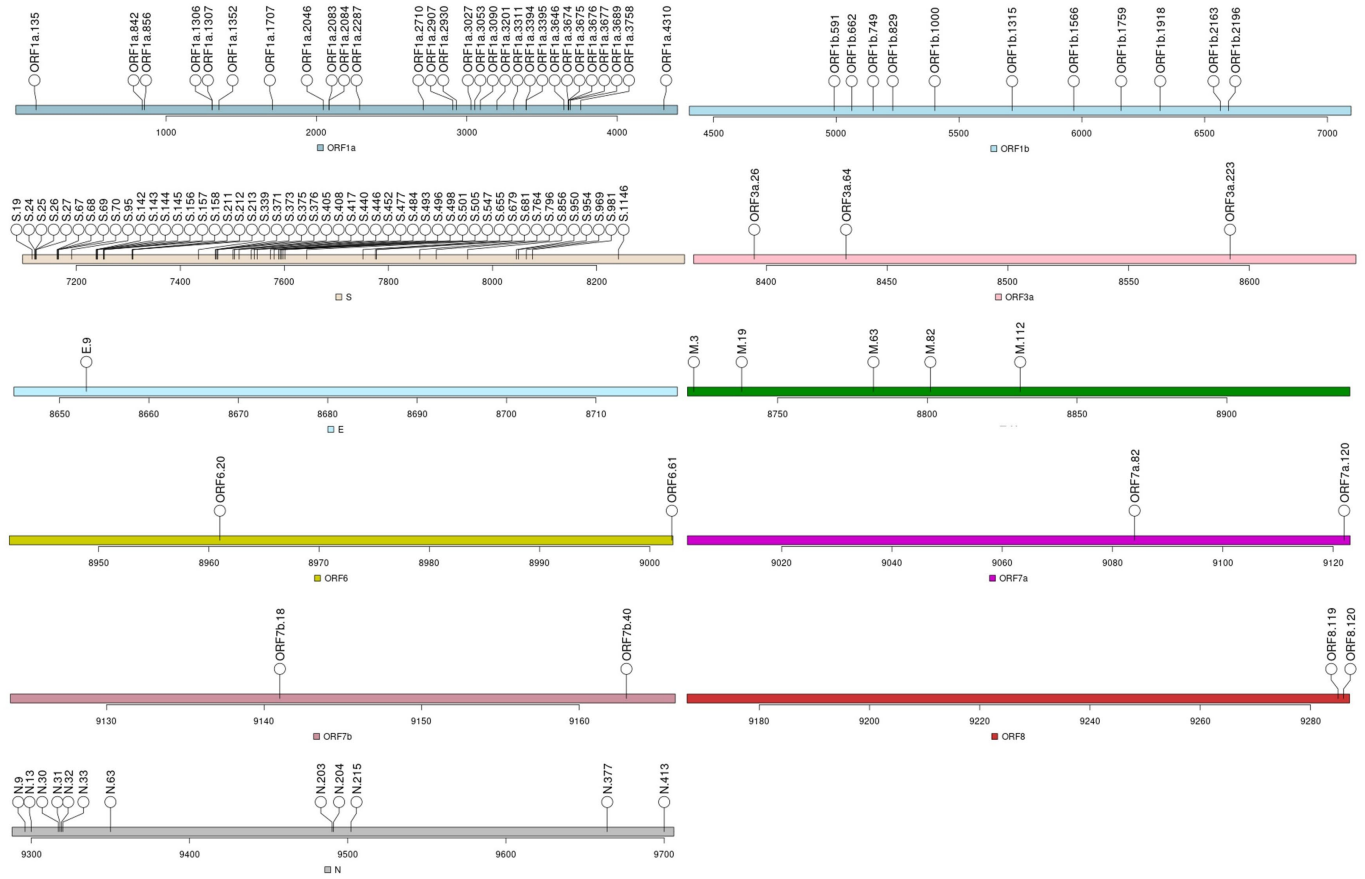
Mutations of interest (MOIs) identified for Dataset_2.

Nine sets of sequences were derived from the 119 MOIs, as it is depicted in Figure 3 and Supplementary Figure S2B. The incidence of SARS-CoV-2 infection for the sets significantly associated with vaccine failure displayed two peaks, in July 2021 and November 2021, during the 21J (Delta) wave (Tables 4, 5).

**Table 4:** Descriptive statistics for non-Omicron sets in Dataset_2 among fully vaccinated and non-fully vaccinated COVID-19 patients.

	Set1_ds2 *n* = 211	Set2_ds2 *n* = 138	Set3_ds2 *n* = 480	Set4_ds2 *n* = 226	Set5_ds2 *n* = 507	Set6_ds2 *n* = 105
Full active immunity, *n*	8	86	176	82	200	39
FDR^a^ value	1.03E-12^b^	5.08E-13	0.529	0.737	0.034	0.818
Clade	20I (Alpha)	21J (Delta)	21J (Delta)	21J (Delta)	21J (Delta)	21J (Delta)
Peak^c^ (*n*)	February 2021 (72)	July 2021 (69)	November 2021 (233)	November 2021 (87)	November 2021 (265)	December 2021 (48)

^a^FDR, False discovery rate.^b^Expected frequency exceeded the observed frequency for fully vaccinated patients infected by SARS-CoV-2 sequences sharing the same combination of MOIs, in clinical terms this translates to vaccine coverage or efficacy.^c^Referred to the highest number of cases.

**Table 5:** Descriptive statistics comparing fully vaccinated and booster-dosed COVID-19 patients with those who are not fully vaccinated for Omicron sets of sequences.

	Set7_ds2 *n* = 685	Set8_ds2 *n* = 1,184	Set9_ds2 *n* = 507
Full active immunity, *n*	137	408	80
FDR^a^ Value	1.96E-16^b^	0.928	2.37E-19^b^
Booster, *n*	121	365	59
FDR Value	3.89E-19^c^	0.074	6.34E-26^c^
Clade	21K (Omicron)	21L (Omicron)	22B (Omicron)
Peak^d^ (*n*)	February 2022 (479)	April 2022 (383)	June 2022 (239)

^a^FDR, False discovery rate.^b^Expected frequency exceeded the observed frequency for fully vaccinated patients infected by SARS-CoV-2 sequences sharing the same combination of MOIs, in clinical terms this translates to vaccine coverage or efficacy.^c^Expected frequency exceeded the observed frequency for booster patients infected by SARS-CoV-2 sequences sharing the same combination of MOIs, in clinical terms this translates to booster dose efficacy.^d^Referred to the highest number of cases.

Additional timeline information for these sequence sets is available in the Supplementary material, provided as images named SetNumber_Dataset_TotalNumber.png.

When comparing both datasets, we identified seven MOIs that were exclusively present in Dataset_1. These included T3255I (ORF1a), E3965E (ORF1a), I4205I (ORF1a), R346T (S), T478K (S), F486V (S), F486P (S), and F8F (ORF3a). Five of these mutations were defining mutations of the Omicron clades (T3255I (ORF1a), R346T (S), T478K (S), F486V (S), F486P (S)), three did not belong to any Nextstrain Clade (E3965E (ORF1a), I4205I (ORF1a), and F8F (ORF3a)), and two were associated with the 21J (Delta) clade (T3255I (ORF1a) and T478K (S)). On the other hand, Dataset_2 exclusively contained seven MOIs, with none of them overlapping with Dataset_1. Among these, three were defining mutations of the 21K (Omicron) clade (T95I (S), V143- (S), and G496S (S)), while four lacked Nextstrain Clade assignments (L829I (ORF1b), Y1759Y (ORF1b), Y145H (S), and Q9L (N)).

### 3.2 Associations with vaccine failure

#### 3.2.1 Dataset_1

When applying a Chi-square test, individuals who were fully vaccinated and infected with non-Omicron variants showed a statistically significant association with vaccine coverage. This trend was observed from Set1_ds1 to Set4_ds1. Thus, variants that peaked in December, January, March, and April and 2021 were strongly linked to vaccine protection (Table 2). However, Omicron cases in sets of sequences, Set5_ds1 (21L, FDR = 1.58 × 10^−41^), Set6_ds1 and Set7_ds1 (22B and 22E, FDR = 1.32 × 10^−122^ and 1.12 × 10^−50^, respectively), as well as Set8_ds1 (22F and 23A, FDR = 1.24 × 10^−88^), displayed a strong association with COVID-19 infection (Table 3; Supplementary Table S1). The booster dose demonstrated a slight improvement in protection against viral infection, with the exception of Set7_ds1. When comparing Set7_ds1 to Set6_ds2 from the same clade, we observed that both shared the same non-defining MOIs A27- (S), I68- (S), R346T (S) and G30- (N), with the only difference being the presence of a ‘T’ at locus 346 in the spike protein (Table 6).

**Table 6:** Set of sequences sharing the same combination of MOIs in Dataset_1 and Dataset_2 for 22B (Omicron) and 22E (Omicron) variants and their distribution based on vaccine brands.

Non-defining MOIs							
		A27- (S)	I68- (S)	R346T (S)	G30- (N)	FDR^a^	FDR^b^
Dataset_1	Set6_ds1	-	-	R	-	1.32E-122	1.18E-118
	Set7_ds1	-	-	T	-	1.12E-50	2.21E-50
Dataset_2	Set9_ds2	-	-	R	-	2.37E-19^c^	6.34E-26^d^

	Fully vaccinated		Booster		Fully vaccinated		Booster	
	Set6_ds1, *n* = 384	Set9_ds2, *n* = 80	Set6_ds1, *n* = 341	Set9_ds2, *n* = 59	Set7_ds1, *n* = 155	Set9_ds2, *n* = 80	Set7_ds1, *n* = 139	Set9_ds2, *n* = 59
BioNTech-Pfizer, *n* (%)	197 (51.3)	73 (91.3)	179 (52.5)	53 (89.8)	73 (47.1)	73 (91.3)	66 (47.5)	53 (89.8)
Moderna-Lonza, *n* (%)	176 (45.8)	2 (2.5)	161 (47.2)	1 (1.7)	78 (50.3)	2 (2.5)	72 (51.8)	1 (1.7)
Oxford-AstraZeneca, *n* (%)	0 (0.0)	4 (5.0)	0 (0.0)	4 (6.8)	0 (0.0)	4 (5.0)	0 (0.0)	4 (6.8)
J&J-Janssen, *n* (%)	11 (2.9)	1 (1.3)	1 (0.3)	1 (1.7)	4 (2.6)	1 (1.3)	1 (0.7)	1 (1.7)
*p* Value	3.45E-15		1.40E-13		5.34E-13		6.65E-11	

^a^False discovery rate value for the comparison of fully vaccinated vs not fully vaccinated COVID-19 patients.^b^False discovery rate value for the comparison of the patients who received booster dose versus not fully vaccinated COVID-19 patients.^c^Expected frequency exceeded the observed frequency for fully vaccinated patients infected by SARS-CoV-2 sequences sharing the same combination of MOIs, in clinical terms this translates to vaccine coverage or efficacy.^d^Expected frequency exceeded the observed frequency for booster patients infected by SARS-CoV-2 sequences sharing the same combination of MOIs, in clinical terms this translates to booster dose efficacy.

#### 3.2.2 Dataset_2

In the 21J (Delta) clade, five distinct sequence sets (Set2_ds2, Set3_ds2, Set4_ds2, Set5_ds2, and Set6_ds2) were identified, all sharing the same residue substitutions in three non-defining mutations: P2046L (ORF1a), P2287S (ORF1a), and R158- (S) (Table 7). However, the distinguishing factor among these sets was the presence of four additional non-defining mutations, specifically L829I (ORF1b), T95I (S), Y145H (S), and Q9L (N) (Table 7). Two of these sets, Set2_ds2 (FDR = 5.08 × 10^−13^) and Set5_ds2 (FDR = 0.034), showed significant associations with low vaccine coverage, although they differed in the defining mutation G142D (S) (Supplementary Figure S2B). Both high-risk sets shared non-defining haplotypes, having a threonine at position 829 in the ORF1a and a leucine at position 9 in the N protein (Supplementary Figure S2B; Table 7). On the other hand, Set1_ds2 (clade 20I, Alpha variant) and two sets of sequences infected by the Omicron variant, namely Set7_ds2 (21K) and Set9_ds2 (22B), exhibited a strong association with reduced susceptibility to SARS-CoV-2 infection.

**Table 7:** Set of sequences sharing the same combination of MOIs in Dataset_1 and Dataset_2 for 21J (Delta) variant and their distribution based on vaccine brands.

Non-defining MOIs									
		P2046L (ORF1a)	P2287S (ORF1a)	L829I (ORF1b)	T95I (S)	Y145H (S)	R158- (S)	Q9L (N)	FDR^a^
Dataset_1	Set1_ds1	L	S	L	T	Y	-	Q	2.00E-13^vc^
Dataset_2	Set2_ds2	L	S	I	T	Y	-	L	5.08E-13
	Set3_ds2	L	S	L	I	Y	-	Q	0.529
	Set4_ds2	L	S	L	T	Y	-	Q	0.737
	Set5_ds2	L	S	I	T	Y	-	L	0.034
	Set6_ds2	L	S	L	I	H	-	Q	0.818

	Set2_ds2, *n* = 86	Set5_ds2, *n* = 200
BioNTech-Pfizer, *n* (%)	74 (86.0)	141 (70.5)
Moderna-Lonza, *n* (%)	0 (0.0)	6 (3.0)
Oxford-AstraZeneca, *n* (%)	11 (12.8)	42 (21.0)
J&J-Janssen, *n* (%)	1 (1.2)	8 (4.0)
Unknown, *n* (%)	0 (0.0)	3 (1.5)
*p* Value	0.049	

^a^False discovery rate value for the comparison of fully vaccinated vs not fully vaccinated COVID-19 patients.

Additionally, it was observed that the booster dose was found to provide increased protection against the Omicron 21K and 22B clades (Table 5; Supplementary Table S1).

### 3.3 Conservation analysis

#### 3.3.1 Dataset_1

The conservation scores, used to assess the overall functionality based on the physicochemical properties of the amino acid substitutions, revealed a strong conservation index in 38.7% of the changes. However, 37.0% of these substitutions exhibited low conservation similarities (Table 8; Supplementary Figure S2A; Supplementary Table S2). These substitutions were predominantly composed of hydrophobic (66.2%) and polar (55.4%) amino acids (Table 8). For a more in-depth analysis of these changes, we grouped the set of sequences associated with vaccine failure caused by the Omicron variant considering clades 21L, 22B/22E and 22F/23A, as this is the only variant that was associated with vaccine failure. The analysis identified 52 loci that differed between Set5_ds1, Set6_ds1, Set7_ds1, and Set8_ds1 resulting in 55 amino acids changes (Supplementary Table S2) possessing mainly hydrophobic (63.6%) and polar (61.8%) properties (Table 8). Of these substitutions, 40.4% showed an intermediate conservation index. Focusing on non-clade-defining MOIs, we identified 11 loci that differed between Set5_ds1, Set6_ds1, Set7_ds1, and Set8_ds1, including L3201F (ORF1a), G662S (ORF1b), V213G/E (S), G339D/H (S), R346T (S), G446S (S), L452R (S), F486V/P (S), Q493R (S), D3N (M) and D61L (ORF6). These differences resulted in 14 amino acid changes, primarily characterized by polar (64.3%) and small (57.7%) properties (Table 8).

**Table 8:** Physicochemical properties of the amino acids substituted in Dataset_1 and Dataset_2.

	Dataset_1 Whole	Dataset_1 Omicron	Dataset_1 Omicron Additional	Dataset_2 Whole	Dataset_2 21J (Delta)	Dataset_2 21J (Delta) Additional
Number of amino acids substitution	74	55	14	81	26	6
Property, *n* (%)						
Hydrophobic	49 (66.2)	35 (63.6)	6 (42.9)	56 (69.1)	18 (69.2)	5 (83.3)
Polar	41 (55.4)	34 (61.8)	9 (64.3)	43 (53.1)	11 (42.3)	2 (33.3)
Small	29 (39.2)	20 (36.4)	8 (57.1)	31 (38.3)	11 (42.3)	1 (16.7)
Proline	2 (2.7)	2 (3.6)	1 (7.1)	1 (1.2)	0 (0.0)	0 (0.0)
Tiny	12 (16.2)	7 (12.7)	3 (21.4)	14 (17.3)	6 (23.1)	1 (16.7)
Aliphatic	18 (24.3)	11 (20.0)	2 (14.3)	22 (27.2)	10 (38.5)	4 (66.7)
Aromatic	13 (17.6)	12 (21.8)	2 (14.3)	14 (17.3)	2 (7.7)	1 (16.7)
Positive	19 (25.7)	17 (30.9)	3 (21.4)	21 (25.9)	4 (15.4)	1 (16.7)
Negative	4 (5.4)	4 (7.3)	2 (14.3)	3 (3.7)	1 (3.8)	0 (0.0)
Charged	23 (31.1)	21 (38.2)	5 (35.7)	24 (29.6)	5 (19.2)	1 (16.7)
Number of MOIs	119	52	11	119	26	6
Conservation Index, value (symbol), *n* (%)						
10 (*)	19 (16.0)	0 (0.0)	0 (0.0)	18 (15.1)	1 (3.8)	1 (16.7)
9 and 8 (:)	27 (22.7)	17 (32.7)	5 (45.5)	29 (24.4)	9 (34.6)	1 (16.7)
7 and 6 (.)	29 (24.4)	21 (40.4)	1 (9.1)	28 (23.5)	10 (38.5)	4 (66.7)
≤ 5 (blank space)	44 (37.0)	14 (26.9)	5 (45.5)	44 (37.0)	6 (23.1)	0 (0.0)

#### 3.3.2 Dataset_2

The conservation index, assessing properties that are positively or negatively conserved, displayed high values in 39.5% of the MOIs, while 37.0% showed low conservation values (Table 8; Supplementary Figure S2B; Supplementary Table S3). The predominant physicochemical properties affected by these changes were hydrophobic (69.1%) and polar (53.1%) (Table 8). Only the 21J variant was found to be associated with vaccine escape so it was analyzed in detail. This analysis identified 26 loci that differed between set of sequences ranging from Set2_ds2 to Set6_ds2 resulting in 26 amino acid changes characterized predominantly by their hydrophobic (69.2%) property (Table 8). Substitutions with a conservation index corresponding to high and intermediate conservation were dominant (34.6 and 38.5%, respectively). Non-defining mutations of 21J (Delta) included P2046L, P2287S, L829I, T95I, Y145H, R158-, and Q9L which were characterized by hydrophobic (83.3%) and aliphatic (66.7%) amino acid substitutions. These changes were mainly led to an intermediate conservation of physicochemical properties.

### 3.4 Vaccine effectiveness

Given the significant role of non-clade-defining mutations in the risk of vaccine failure, the next step was to conduct a joint analysis of both datasets. This analysis aimed to identify non-clade defining MOIs associated with vaccine failure caused by the Omicron (21L, 22B and 22E) and Delta (21J) clades, which are common variants present in both datasets but were not initially linked to vaccine failure in the same manner.

Both set5_ds1 and set8_ds2 belonged to the 21L clade and shared three additional mutations: L3201F (ORF1a), A27- (S), and G30- (S) (Table 9). However, while set5 was associated with vaccine failure even after receiving a booster dose (FDR = 1.58 × 10^−41^ and 3.20 × 10^−31^, respectively), set8_ds2 did not exhibit the same level of association. The Chi-Square test revealed statistically significant differences in vaccine distribution between fully vaccinated individuals (FDR = 3.58 × 10^−17^) and those who received a booster dose (FDR = 1.71 × 10^−20^) when comparing set5_ds1 and set8_ds2, respectively. Set5_ds1 was primarily associated with the Pfizer and Moderna vaccines (61.1 and 35.4% for fully vaccinated, and 59.3 and 39.0% for booster doses, respectively). In contrast, set8_ds2 was predominantly associated with Pfizer and AstraZeneca vaccines (77.9 and 13.6% for fully vaccinated, and 78.9 and 14.5% for booster doses, respectively). Furthermore, the analysis of the genetic distance in the sets of sequences showed remarkable differences between sets, with the genetic distance of set8_ds2 being 3.8 times higher compared to set5_ds (Figure 3).

**Table 9:** Set of sequences sharing the same combination of MOIs in Dataset_1 and Dataset_2 for 21L (Omicron) variant and their distribution based on vaccine brands.

Non-defining MOIs						
		L3201F (ORF1a)	A27- (S)	G30- (N)	FDR^a^	FDR^b^
Dataset_1	Set5_ds1	F	-	-	1.58E-41	3.20E-31
Dataset_2	Set8_ds2	F	-	-	0.928	0.074

	Fully vaccinated		Booster	
	Set5_ds1, *n* = 144	Set8_ds2, *n* = 408	Set5_ds1, *n* = 118	Set8_ds2, *n* = 365
BioNTech-Pfizer, *n* (%)	88 (61.1)	318 (77.9)	70 (59.3)	288 (78.9)
Moderna-Lonza, *n* (%)	51 (35.4)	27 (6.6)	46 (39.0)	20 (5.5)
Oxford-AstraZeneca, *n* (%)	2 (1.4)	55 (13.5)	0 (0.0)	53 (14.5)
J&J-Janssen, *n* (%)	3 (2.1)	5 (1)	2 (1.7)	1 (0.3)
Unknown, *n* (%)	0 (0.0)	3 (0.7)	0 (0.0)	3 (0.8)
*p* value	3.58E-17		1.71E-20	

^a^False discovery rate value for the comparison of fully vaccinated vs not fully vaccinated COVID-19 patients.^b^False discovery rate value for the comparison of the patients who received booster dose versus not fully vaccinated COVID-19 patients.

**Figure 4:**
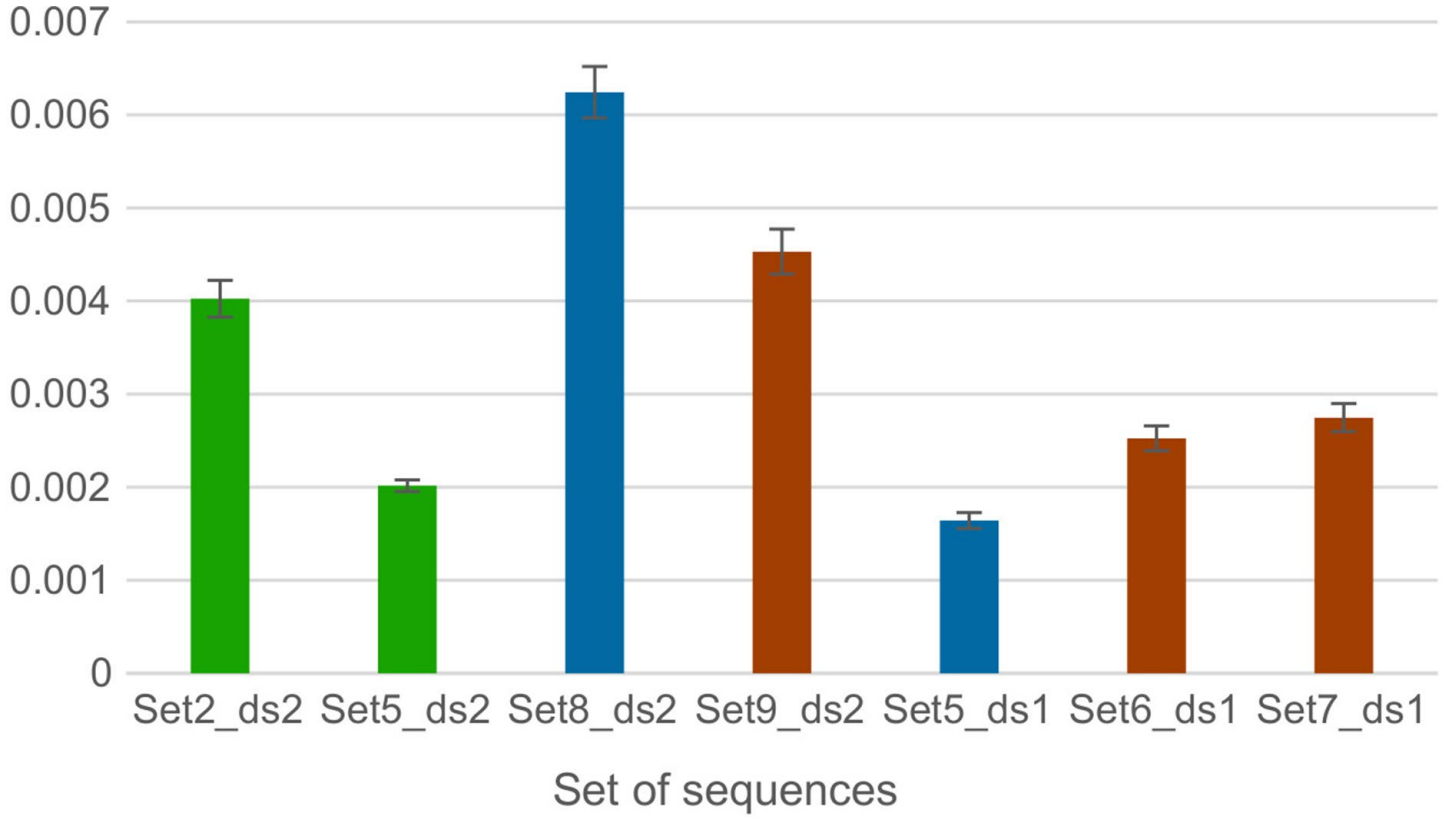
Genetic distances between amino acids per site within each sequence set of Dataset 1 and 2 obtained by averaging all sequence pairs, along with the standard error estimates The average distance was calculated using the Bootstrap method for variance estimation, with 1,000 bootstrap replicates. The p-distance model was used for the amino acid substitution type. Ambiguous positions were removed for each pair of sequences using the pairwise deletion option. The bars are organized based on the datasets and are color-coded according to sets of sequences that share the same MOIs.

For the sets of sequences isolated from COVID-19 patients infected with clades 22B and 22E of the Omicron variant (Table 6), set6_ds1 and set7_ds1 (Dataset_1) and set9_ds2 (Datataset_2) shared the same non-clade defining mutations in the spike (A27-, I68- and R346T) and nucleocapsid (G30-). However, from Dataset_1 only two sequence sets corresponding to fully vaccinated patients (set6_ds1 FDR = 1.32 × 10^−122^; set7_ds1 FDR = 1.12 × 10^−50^) and patients who received the third dose (set6_ds1 FDR = 1.18 × 10^−118^; set7_ds1 FDR = 2.21 × 10^−50^) were associated with vaccine failure. Fully vaccinated patients from Dataset_1 primarily received Pfizer (51.3% for set6_ds1 and 47.1% for set7_ds1) and Moderna (45.8% for set6_ds1 and 50.3% for set7_ds1) vaccines, in contrast to Dataset_2, where 91.3% of patients received Pfizer. Statistically significant differences were found when comparing the FV distribution of these sets (FDR = 3.45 × 10^−15^ for set6_ds1 vs. set9_ds2 and 5.34 × 10^−13^ for set7_ds1 vs. set9_ds2, respectively). For patients who received a booster dose, the main vaccine brands and their associations with vaccine failure remained consistent (FDR = 1.40 × 10^−13^ (set6_ds1 vs. set9_ds2); FDR = 6.65 × 10–11 (set7_ds1 vs. set9_ds2)). Set6_ds1 and set7_ds1 displayed comparable genetic distances among sequences. However, set9_ds2 exhibited a higher genetic distance (Figure 4).

In both datasets, the set of sequences identified as 21J (Delta) shared three additional MOIs, specifically P2046L (ORF1a), P2287S (ORF1a) and R158- (S) (Table 7). Dataset_2 exclusively featured four mutations including L829I (ORF1b), T95I (S), Y145H (S) and Q9L (N). In contrast, these mutations did not meet the MOI criteria for Dataset_2, bearing the wild type amino acids.

Only Set2_ds2 and Set5_ds2 from Dataset_2 were associated with vaccine failure. The vaccine distribution between these risk sets exhibited marginal significance (FDR = 0.049), with the major vaccine brands being both Pfizer and AstraZeneca. The genetic distances for set2_ds2 were twice as high as those observed in set5_ds2 (Figure 4).

There was no correlation between the variability of amino acids in the sets of sequences, measured by the genetic distance, and the loss of vaccine effectiveness for the compared clades.

## 4 Discussion

Mutations and deletions in SARS-CoV-2 proteins can significantly alter their structure and function. This study investigated the impact of mutations of interest (MOI) across the entire SARS-CoV-2 proteome on vaccine escape using two datasets. The analysis revealed several mutations and combinations of residues that may influence vaccine coverage, particularly concerning the Delta (clade 21J) and Omicron BA.2 (clade 21L) variants.

For the Delta variant, mutations at P2046L (ORF1a), P2287S (ORF1a), L829I (ORF1b), R158- (S) and Q9L (N) were identified as potentially crucial for vaccine effectiveness. Similarly, mutations such as L3201F (ORF1a), A27- (S) and G30- (N) in the Omicron variant were found to impact vaccine effectiveness. However, deletions A27- (S), I68- (S) and G30- (N), along with the R346T mutation in the spike protein of the Omicron variant, may further compromise vaccine effectiveness.

Observations of mutations in the spike protein, particularly A27-, I68-, and R158- located within the N-terminal domain (NTD), and the R346T mutation within the Receptor Binding Domain (RBD), align with previous studies. Molecular dynamics simulations have shown the critical involvement of NTD residues in interactions with monoclonal antibodies (42), suggesting potential immune evasion risks for viruses carrying mutations in these regions. Furthermore, mutations within the RBD have been shown to affect the binding affinity to the ACE2 receptor, indicating potential shifts in the binding free energy of the RBD-ACE2 complex and modified chemical interactions, leading to increased stability (43).

Limited published data on vaccine efficacy or immunity related to the A27-(S) deletion were found. However, the spike 68–76 deletion within the NTD was identified in a human hepatoma cell clone termed Huh7.5-adapted-SARS2, indicating genetic adaptations. This modified version of SARS-CoV-2 effectively infiltrated A549 lung cancer cells, inducing cellular damage, a capability absent in the original strain, which exhibited no infectivity toward A549 cells. Additionally, the Spike 68–76 deletion variant displayed increased susceptibility to IFN-α2b treatment in comparison to the wild-type SARS-CoV-2 strain. However, the Spike 68–76 deletion was not found in SARS-CoV-2 isolates obtained from VERO E6 cells (44). In the context of vaccine stability and effectiveness, it suggests that despite the presence of the 68–76 deletion in vaccine batches (CoronaVac), it might not drastically alter the vaccine’s effectiveness. The R158- (S) mutation in combination with E156G/157 deletion and L452R mutation has been suggested to exhibit higher infectivity in spike-pseudotyped viruses (45). However, experimental evidence points to the 156–158 deletion notably diminishing the neutralization capacity against antibodies present in the sera of convalescent COVID-19 patients and vaccinated individuals (42, 46, 47).

The R346T change in the RBD is a key mutation for neutralization escape, enhanced fusogenicity, and enhanced S protein processing. Structural modeling suggests that R346T appears to disrupt salt bridge formation between the S protein and class III monoclonal antibodies (e.g., Cilgavimab), lowering effectiveness (48, 49). However, in our study the set of sequences containing R indicates poorer vaccine coverage than T for the Omicron clades 22B/22E.

The outcomes related to spike mutations impacting the infectivity of SARS-CoV-2 appear to exhibit a wide-ranging scope. Conversely, the available data regarding the influence of mutations occurring in other viral proteins seems comparatively constrained.

An analysis conducted on 244 SARS-CoV-2 positive samples, gathered during the second wave of the pandemic, indicated that mutations P2046L and P2287S in the nsp3 (ORF1a) gene might contribute to persistent symptomatic COVID-19 infections post-vaccination (50). Additionally, an investigation involving severe, moderate, and mild COVID-19 cases, encompassing individuals who were either partially or fully vaccinated (with Covishield/Covaxin) or unvaccinated, revealed a marginal association of the P2287S mutation with disease severity (51). The nsp3 protein in the SARS-CoV-2 virus constitutes a crucial component of the viral replicase complex, contributing significantly to multiple functions associated with viral replication (52), transcription (53), and modulation of the host immune response (54), but no specific data on the impact of the L3201F mutation on these functions have been found in the literature.

A study aimed at modeling the fitness of several SARS-CoV-2 lineages by combining the effect of individual mutations introduced a scalable hierarchical Bayesian regression model to analyze all available SARS-CoV-2 genomes. The study identified the L829I (ORF1b) mutation in nsp12, which promotes an amino acid change in the RdRp (RNA-dependent RNA polymerase) thumb subdomain that could affect the function of the enzyme. RdRp plays a critical role in replicating and transcribing the viral genome in RNA viruses like SARS-CoV-2 (55).

The N protein consists of different structural components, namely, an N-arm, an N-terminal RNA-binding domain, a linker region containing serine/arginine-rich loops (SR-rich region), a C-terminal RNA-binding domain, and a C-tail (56–61). Some regions of the N-arm (amino acids 1–46) have been identified as immunodominant epitopes. Specifically, five monoclonal antibodies were developed through a study of epitopes targeting the N protein of SARS-CoV-2. The research revealed that one particular antibody had a specific affinity for the N-arm region of the N protein (62), suggesting the possible involvement of mutations identified in this study such as Q9L and G30- in immune evasion.

As mentioned, some of the substitutions and deletions associated with vaccine failure in our study seem to be in line with previous studies. However, others have not been previously linked to vaccine coverage or immunity. Therefore, a genome-wide analysis of SARS-CoV-2 mutations, and their effects on the proteome, could help to understand the molecular basis of viral vaccine escape, in connection with vaccine and therapeutic drug development.

The effect of COVID-19 booster-dose vaccination against the Omicron variant has been reviewed by a study that identified a total of 27 published studies supporting the effectiveness of booster dose vaccine (63). Our results are consistent with the improved effectiveness of the booster dose against the Omicron variant in Dataset_2, where the vaccines show high coverage further improved by the administration of the third dose for clades 21L and 22B. However, in Dataset_1 where the vaccine is not effective for Omicron clades, administration of the third dose showed a slight protection for clades 21L, 22B/22E, and 22F/23A. We hypothesize that the discrepancies observed between these datasets might be attributed to the molecular composition of the administered vaccines.

Full implementation of SARS-CoV-2 vaccines is a major goal facing the COVID-19 pandemic. A comparative analysis of COVID-19 vaccine characteristics, adverse events, efficacy and effectiveness reported that all vaccines up to 22nd September 2021 appeared to be safe and effective tools against all variants of concern to prevent severe COVID-19, hospitalization, and death. However, the evidence varies greatly depending on the vaccines considered (64). In addition, the effectiveness of BNT162b2/Comirnaty vaccine (Pfizer) against the Omicron variant has been reported as 60% (65). Conversely, the effectiveness of the Spikevax/mRNA-1273 vaccine (Moderna) was published for symptomatic and asymptomatic cases, without specifying effectiveness for different clades of SARS-CoV-2 (66–68). In this line, in our study, the set of sequences associated with vaccine failure were mainly related to nucleic acid-based vaccines developed by BioNTech-Pfizer and Moderna-Lonza. However, Omicron cases where the vaccine exhibited a protective effect showed a high percentage of the Pfizer brand.

Investigation of the physicochemical attributes of amino acids enables an understanding of the intricate dynamics between viral proteins and the host immune system. This exploration provides valuable insights into viral pathogenicity, contributes to vaccine design and shapes strategies for drug development. In our study a significant number of substitutions, evaluated by conservation scores, showed a robust conservation index, indicating a strong correlation with amino acid physicochemical properties in approximately one-third of the changes. Furthermore, a comparable proportion of these substitutions exhibited lower conserved similarities in both datasets, implying an equal prevalence of such disparities among substitutions analyzed using conservation scores. Analysis of the physicochemical properties of amino acid changes revealed a predominant occurrence of hydrophobic and polar amino acid substitutions in both datasets. Substitutions in the Omicron variant (clades 21L, 22B/22E, and 22F/23A) were predominantly characterized by hydrophobic and polar properties. However, the non-defining mutations of each Omicron clade were mainly polar and small. The 21J (Delta) clade sequence set featured mainly amino acid substitutions with hydrophobic properties. The non-defining mutations of 21J (Delta) were distinguished by prevalent hydrophobic and aliphatic amino acid substitutions. This is agreed with the research that showed the significant role of hydrophobic residues in the spike protein, enhancing interactions in the Delta variant (69). Recently, it was highlighted that the defining mutations in the Delta and Omicron variants markedly impact hydrophobicity, polarity, and charge distribution in all regions of the N-protein (70).

The role of missense mutations and deletions in SARS-CoV-2 has been recognized as pivotal for vaccine effectiveness and residue interactions, highlighting the need to elucidate the molecular basis of these substitutions and deletions for advancing vaccine and drug development. Our investigation identified six mutations significantly associated with reduced vaccine coverage, such as P2046L (ORF1a), P2287S (ORF1a), L3201F (ORF1a), L829I (ORF1b), R346T (S), and Q9L (N), along with the four deletions A27- (S), I68- (S), R158- (S), and G30- (N). Analysis of whole proteome sequences of SARS-CoV-2 derived from COVID-19 patients revealed a correlation between non-clade-defining mutations and vaccine effectiveness. Currently approved vaccines primarily target the spike protein. Thus, changes in this protein could challenge vaccine effectiveness. Our findings support a proteome perspective in SARS-CoV-2 vaccine design, which could improve vaccine effectiveness. In addition, we found that amino acid substitutions exhibited predominantly hydrophobic and polar properties. Understanding the physicochemical properties of amino acid substitutions is crucial, as it reveals how these modifications affect protein structure, function, and interactions. This understanding provides valuable insights into disease mechanisms and the identification of potential therapeutic targets.

## Data Availability

The original contributions presented in the study are included in the article/Supplementary material, further inquiries can be directed to the corresponding author.

## Footnotes

^1^https://covariants.org/
