# Supplementary figures and images for: Unlocking the puzzle: non-defining mutations in SARS-CoV-2 proteome may affect vaccine effectiveness
Source: Front Public Health. 2024 Aug 15;12:1386596. doi: 10.3389/fpubh.2024.1386596 (PMC11369981; doi:10.3389/fpubh.2024.1386596)

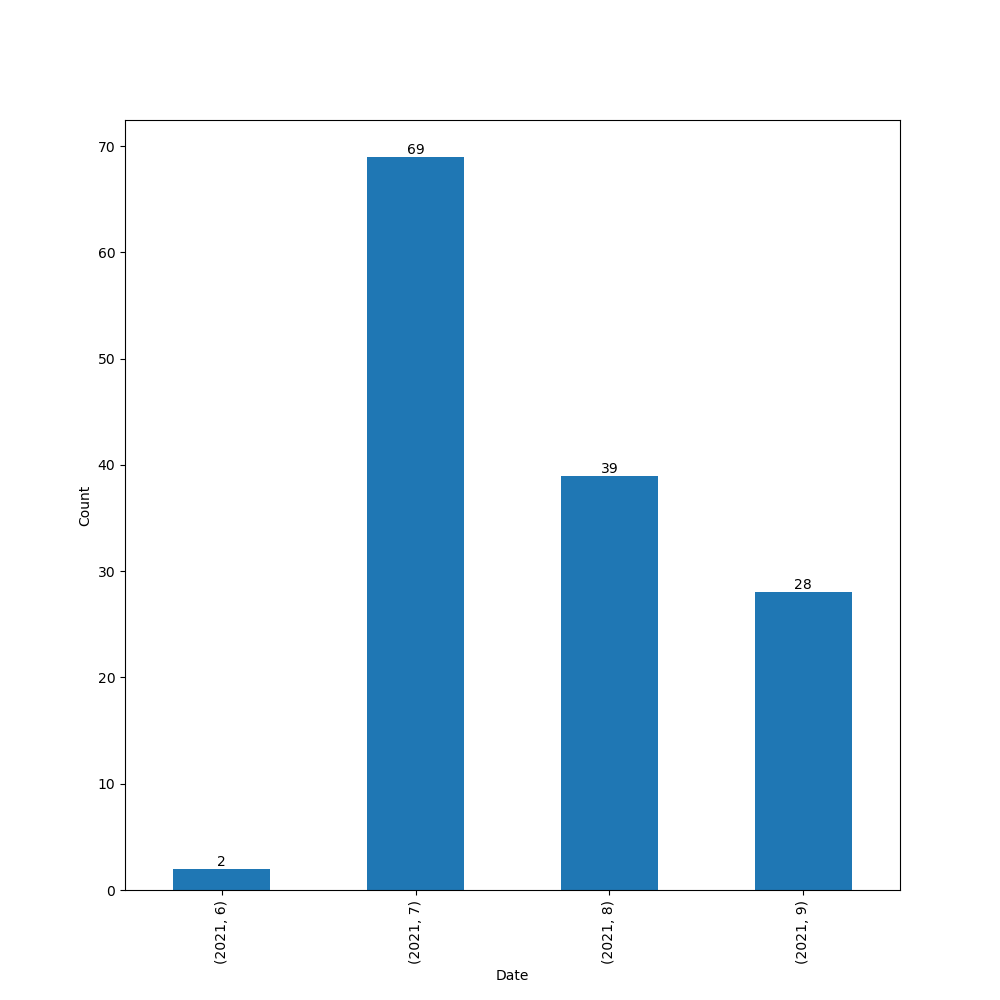

Supplement: Supplementary file 4 [file Image_1.PNG]

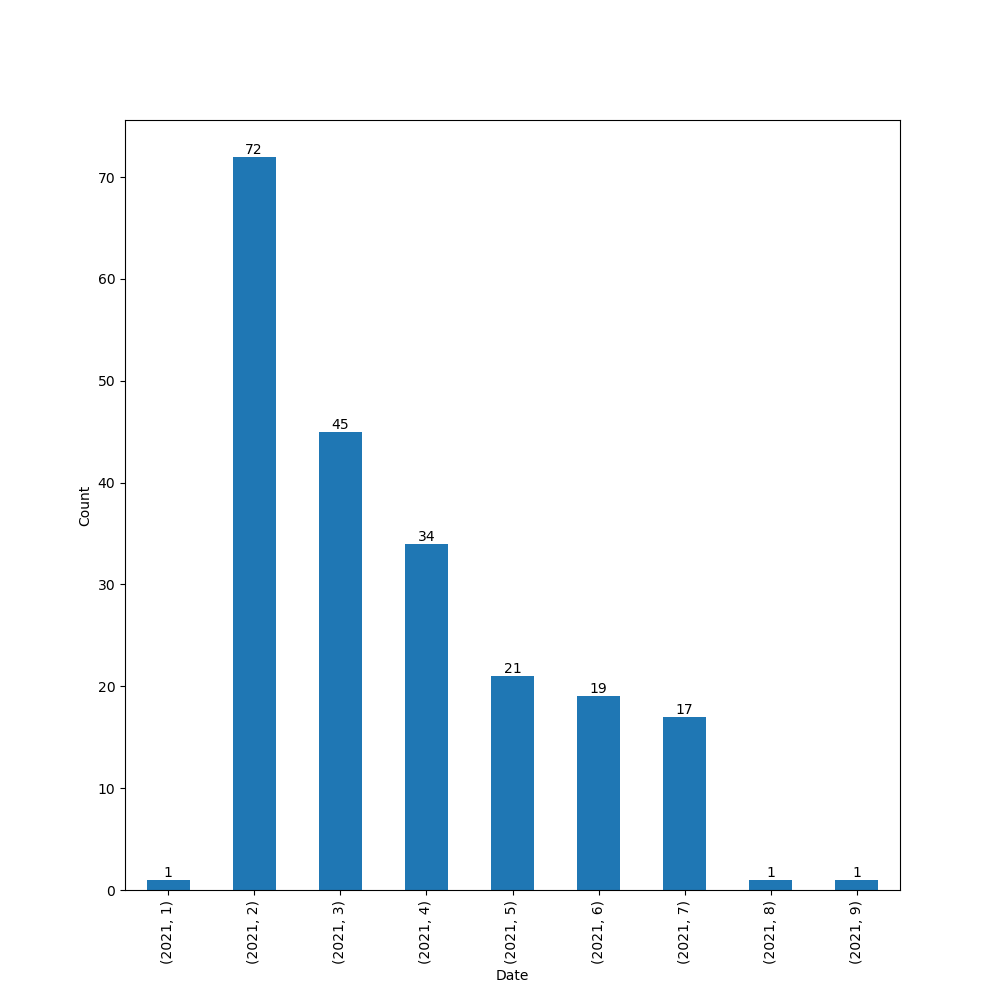

Supplement: Supplementary file 5 [file Image_2.PNG]

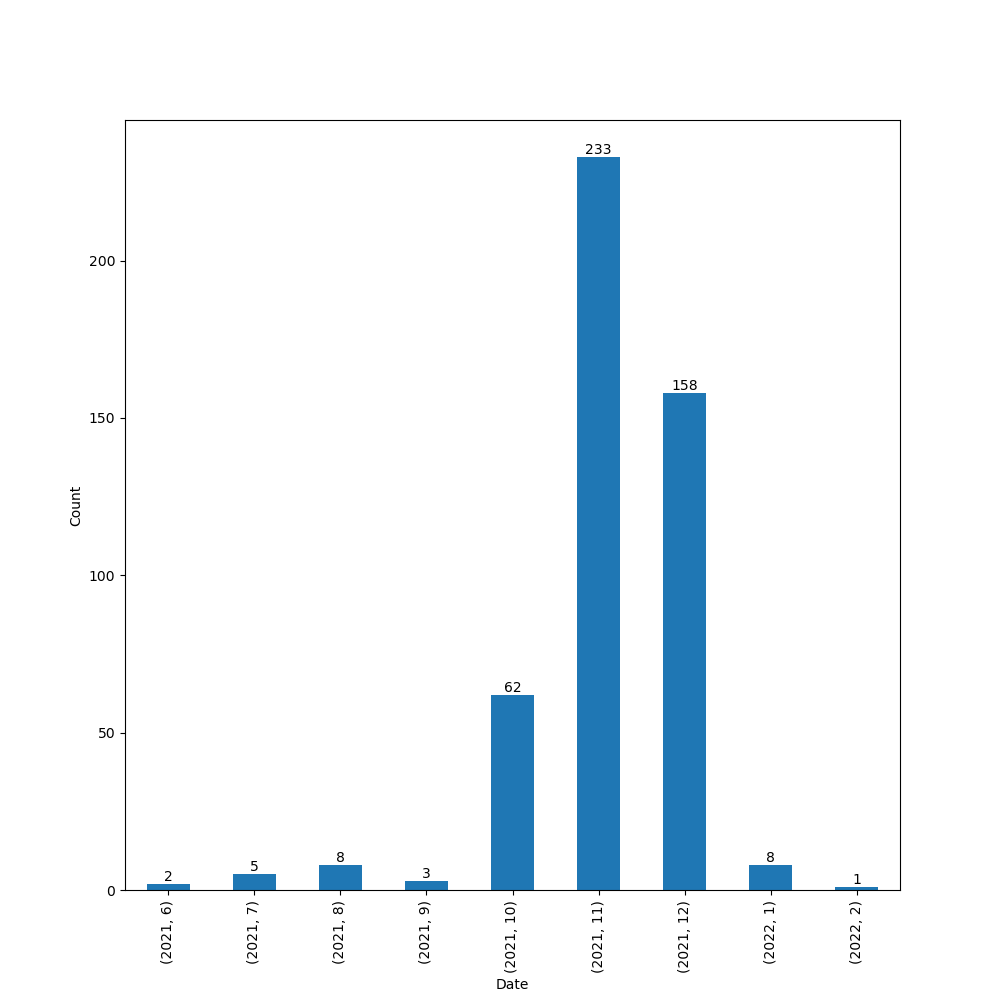

Supplement: Supplementary file 6 [file Image_3.PNG]

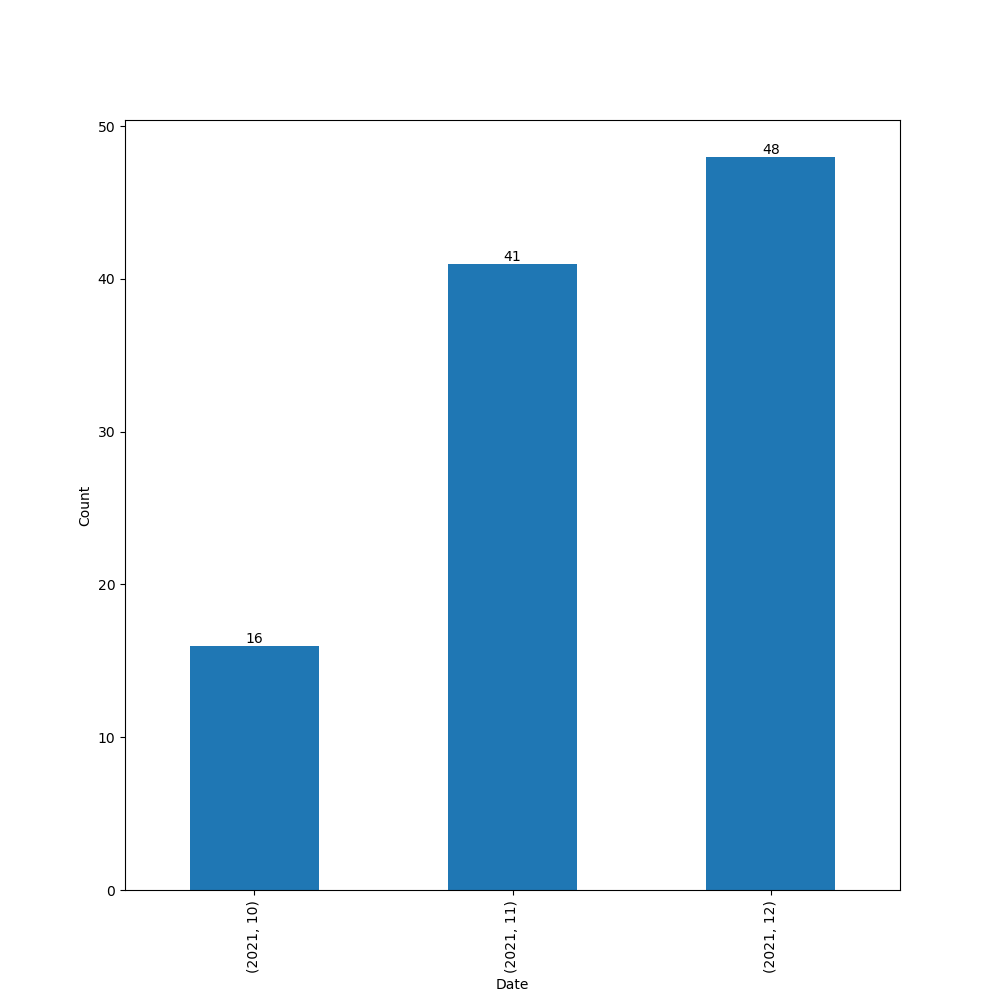

Supplement: Supplementary file 7 [file Image_4.PNG]

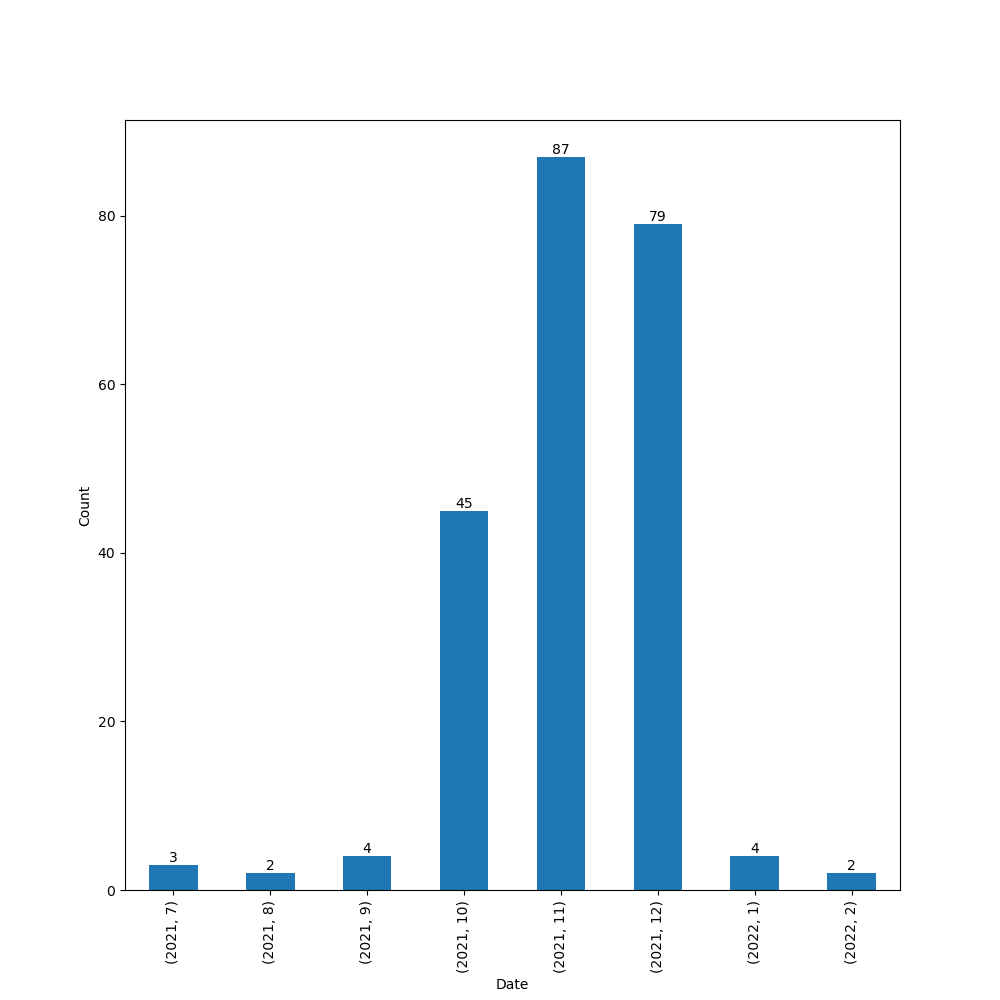

Supplement: Supplementary file 8 [file Image_5.PNG]

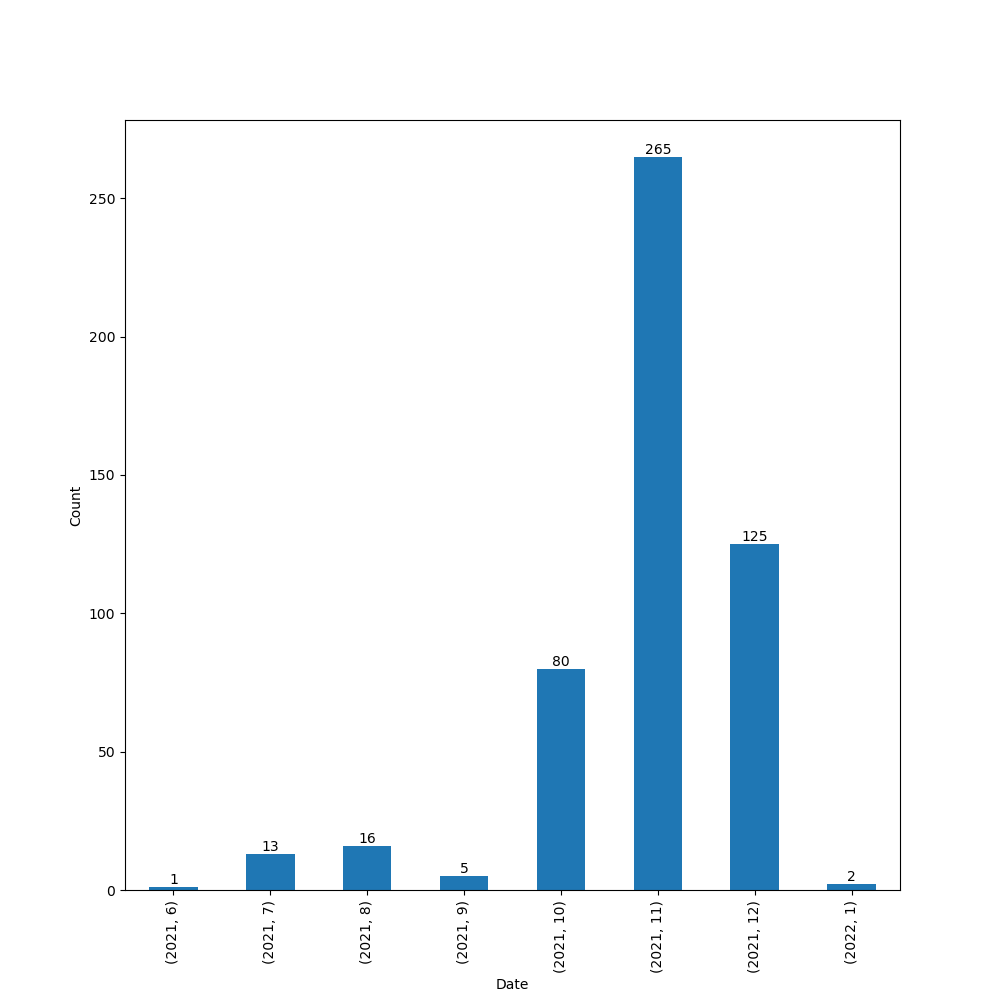

Supplement: Supplementary file 9 [file Image_6.PNG]

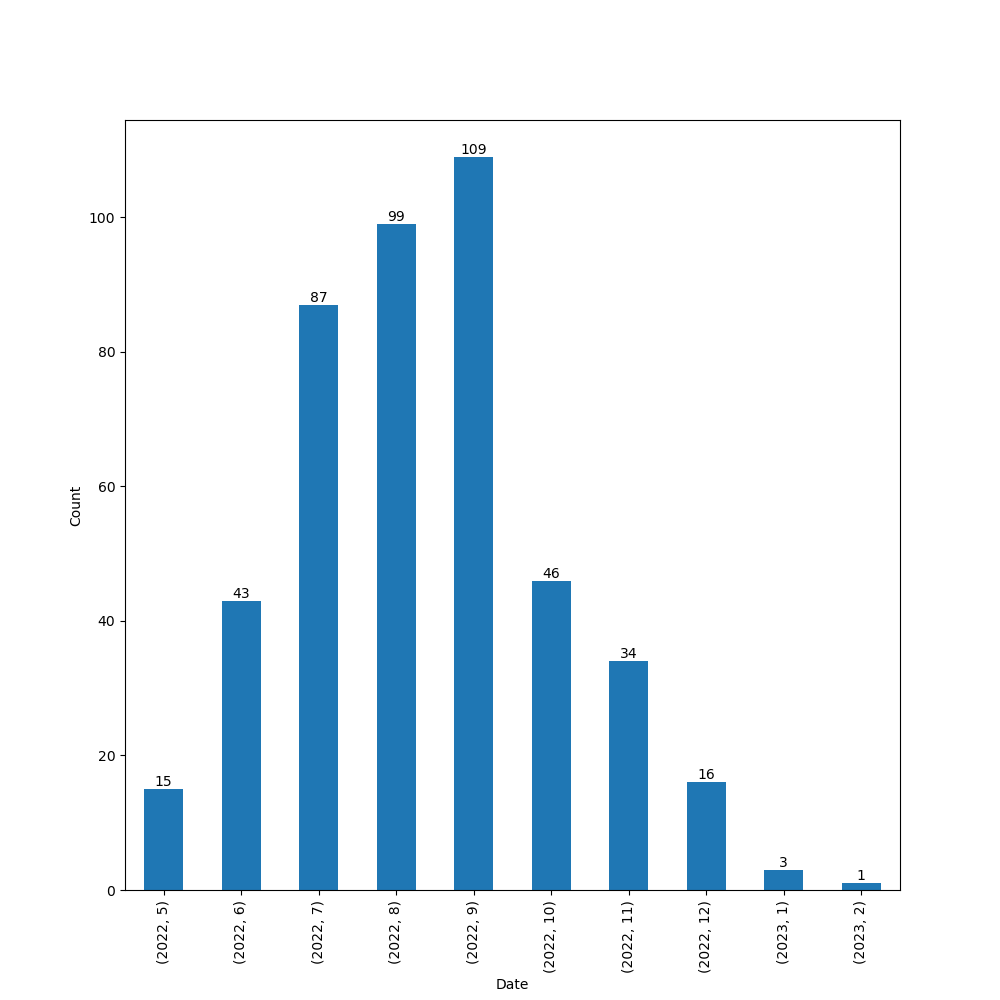

Supplement: Supplementary file 10 [file Image_7.PNG]

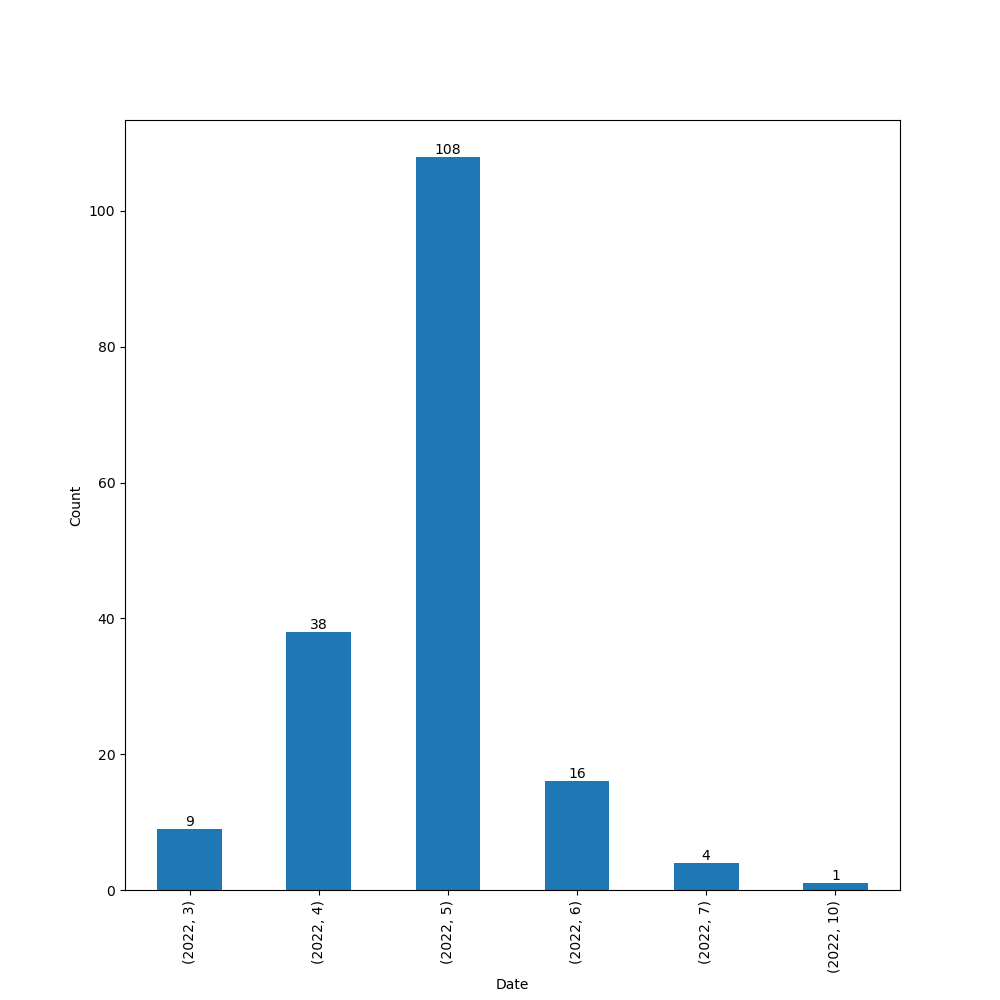

Supplement: Supplementary file 11 [file Image_8.PNG]

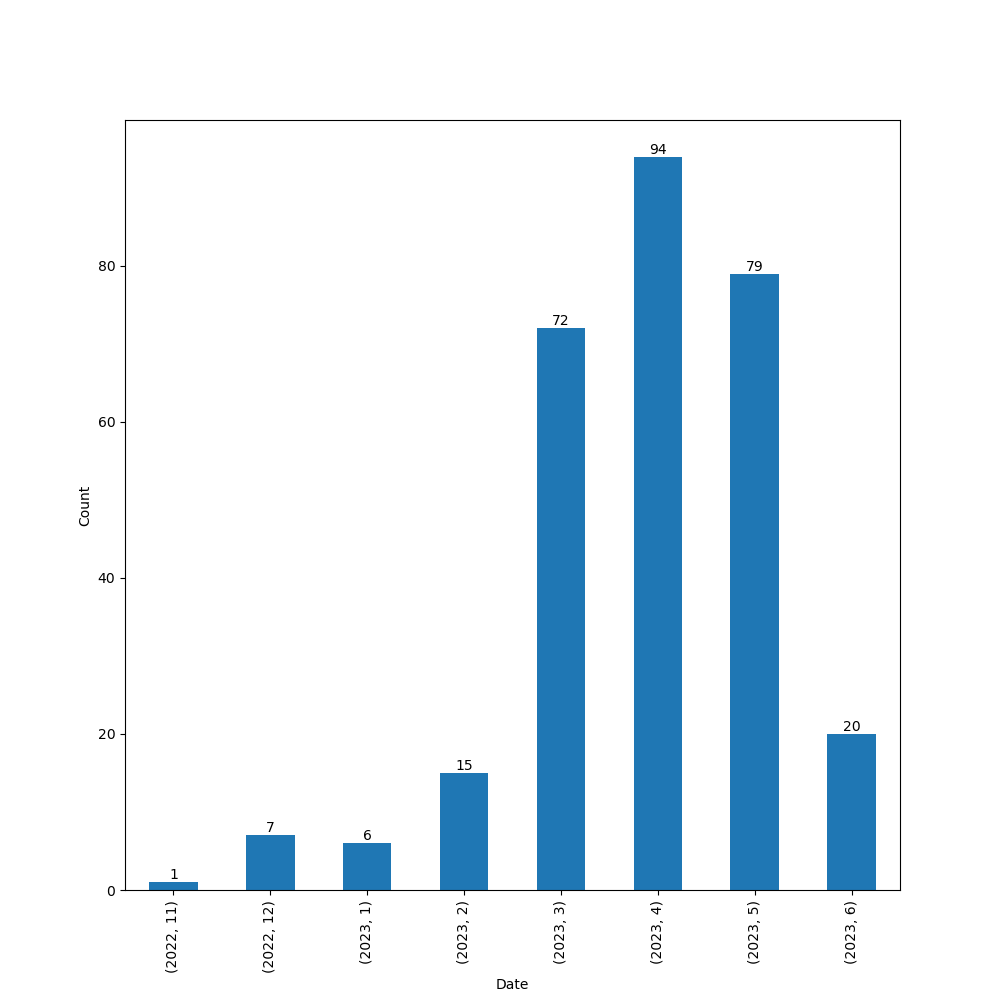

Supplement: Supplementary file 12 [file Image_9.PNG]

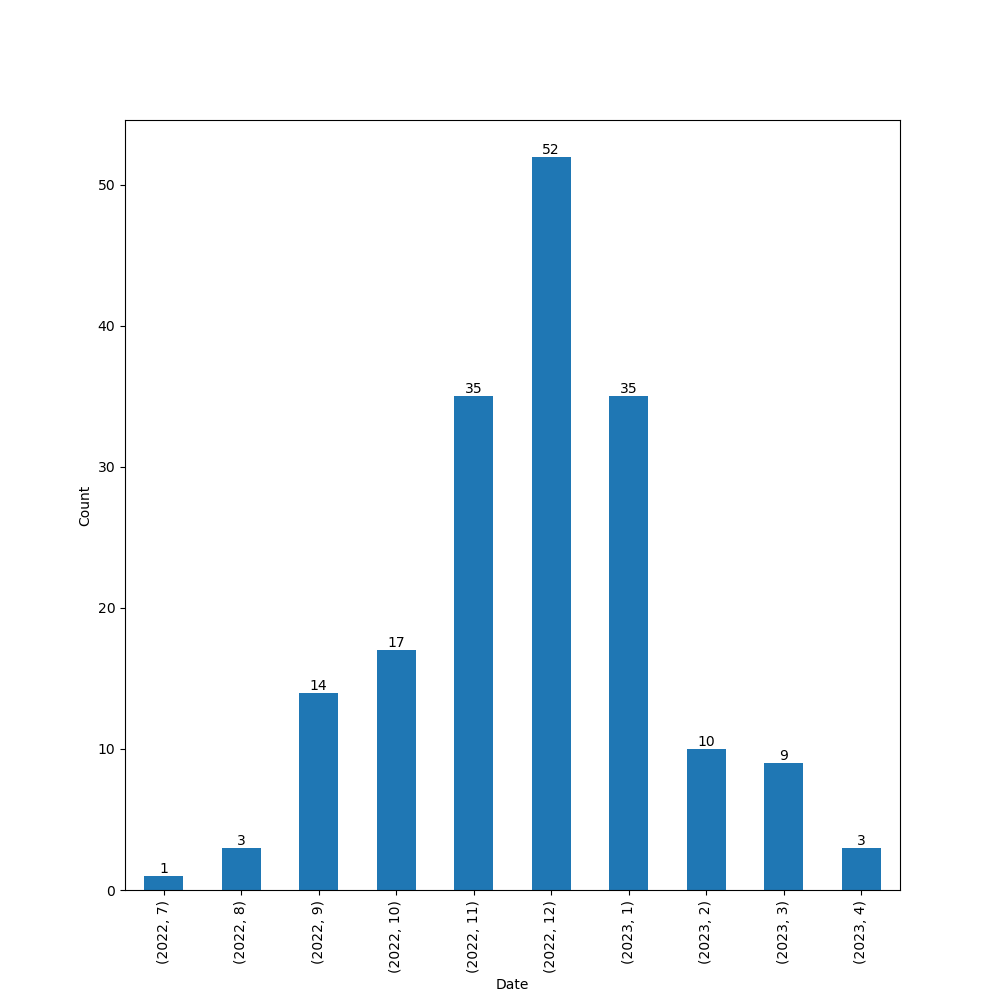

Supplement: Supplementary file 13 [file Image_10.PNG]

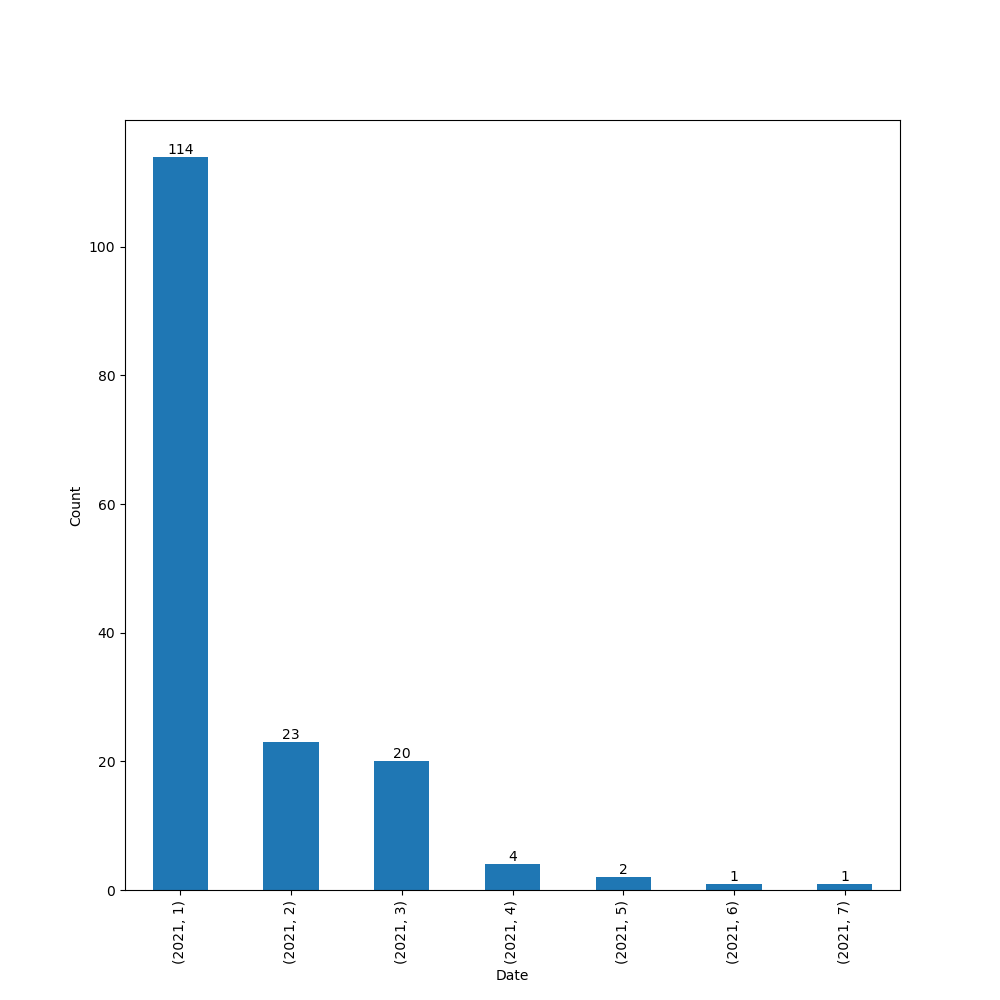

Supplement: Supplementary file 14 [file Image_11.PNG]

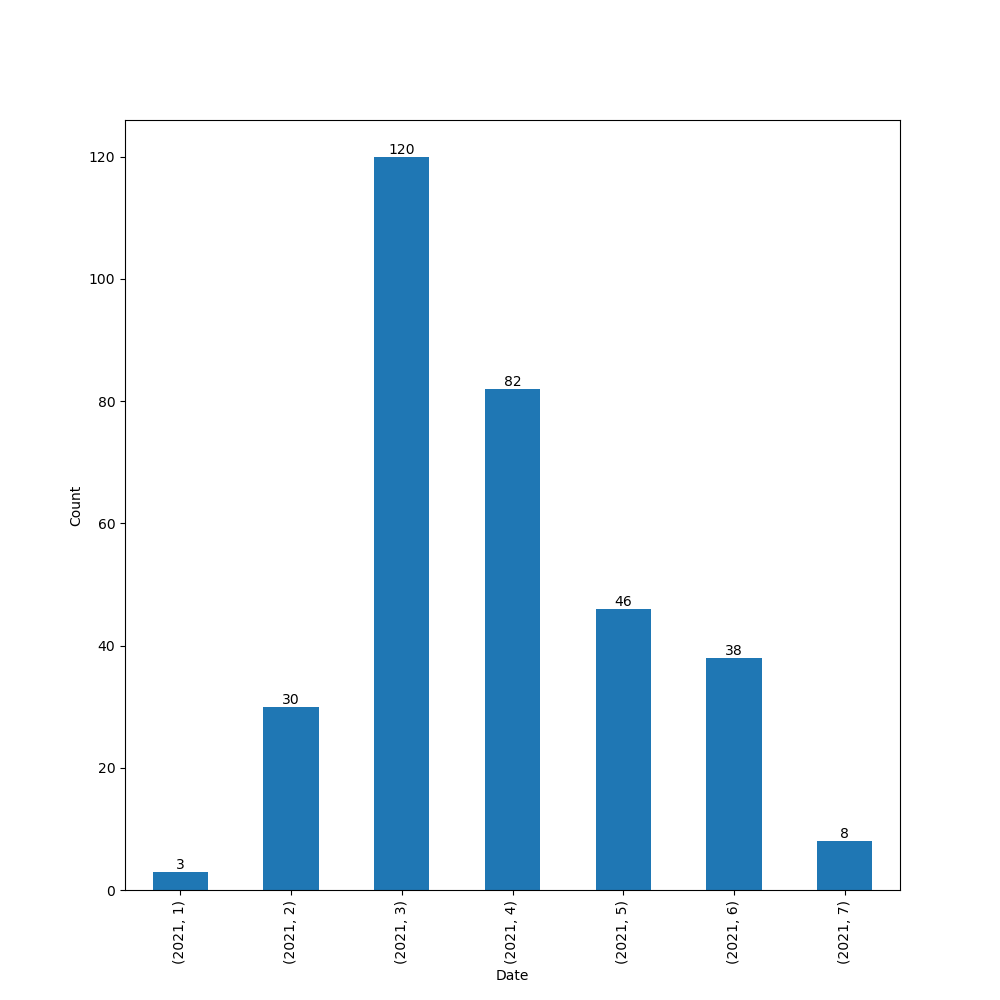

Supplement: Supplementary file 15 [file Image_12.PNG]

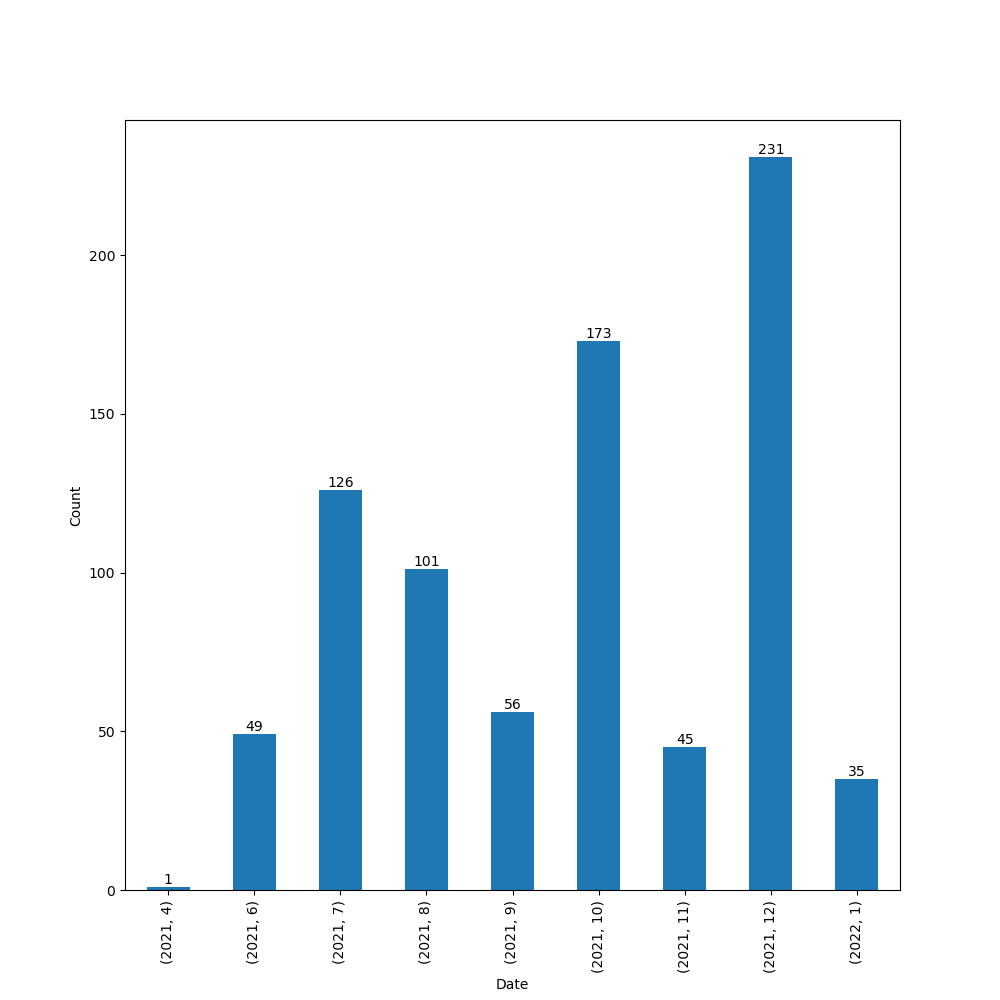

Supplement: Supplementary file 16 [file Image_13.PNG]

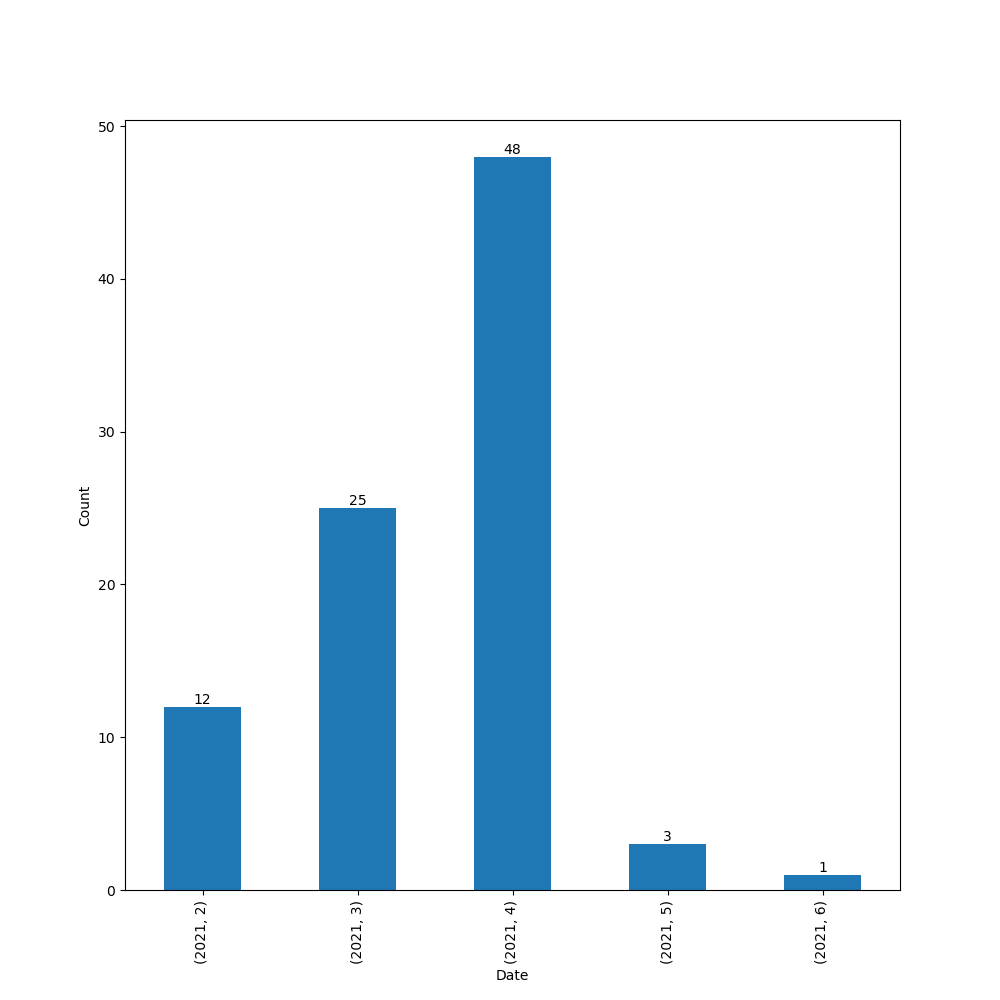

Supplement: Supplementary file 17 [file Image_14.PNG]

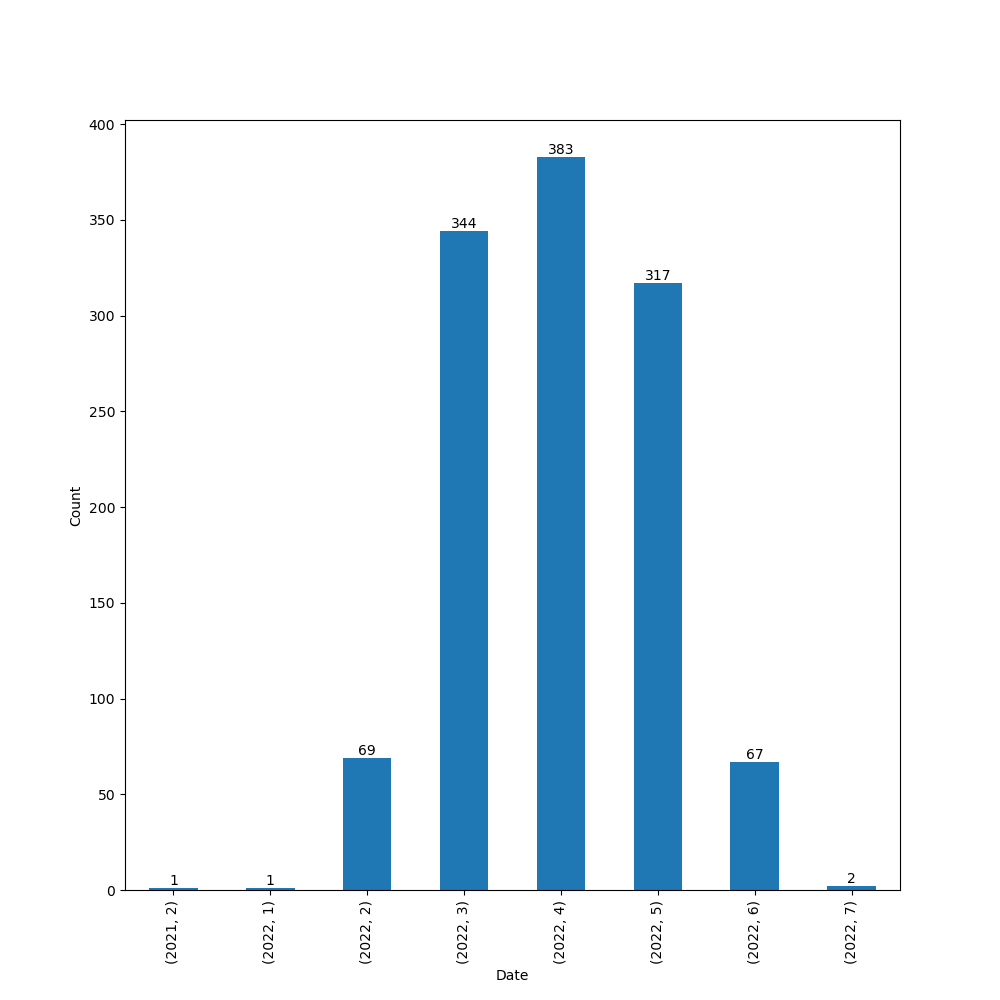

Supplement: Supplementary file 18 [file Image_15.PNG]
