# Supplementary material for: Unlocking the puzzle: non-defining mutations in SARS-CoV-2 proteome may affect vaccine effectiveness
Source: Front Public Health. 2024 Aug 15;12:1386596. doi: 10.3389/fpubh.2024.1386596 (PMC11369981; doi:10.3389/fpubh.2024.1386596)
Supplement: Supplementary file 21 [file Data_Sheet_3.docx]

(A)


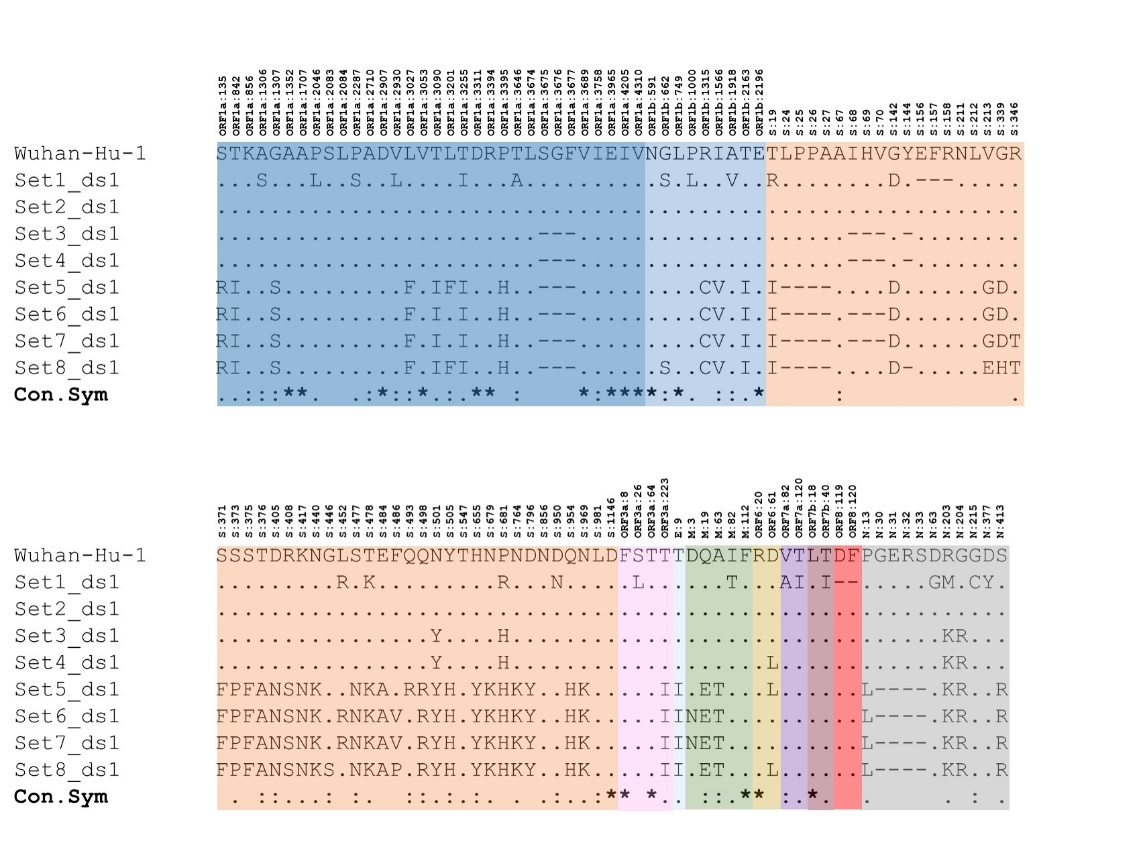


(B)


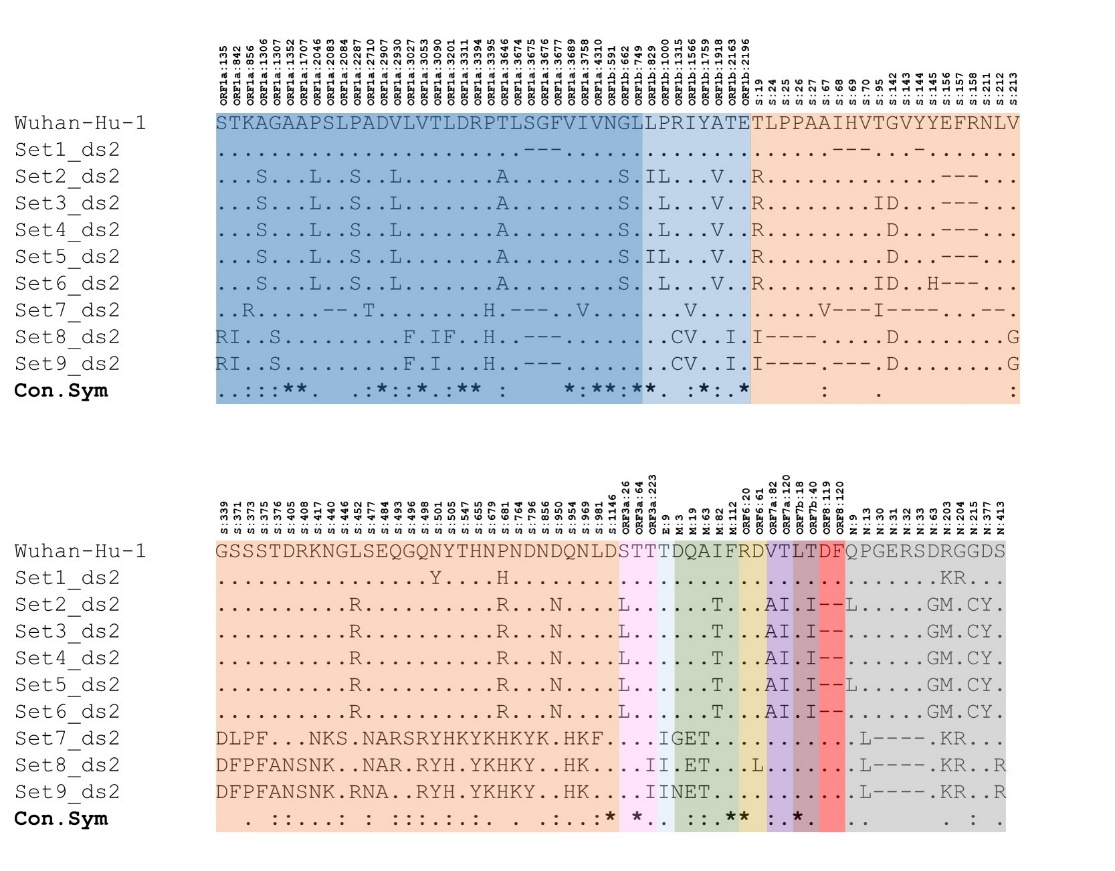


**Supplementary Figure 2.** Represents the sets of sequences with a frequency ≥ 1 % in Panel (A) Dataset_1 and Panel (B) Dataset_2

Mutations are represented in mega non-interleaved format (dots represent amino acids in the Wuhan sequence, while hyphens indicate deletions). MOIs are labeled on the top. Conservation symbols (Con. Sym) are follows: asterisk (*) indicates positions which have a single, fully conserved residue (conservation number 10); colon (:) indicates conservation between amino acids of strongly similar properties (conservation number 9 or 8); period (.) indicates conservation between amino acids of weakly similar properties (conservation number 7 or 6). A space indicates a conservation number ≤ 5.
